# Inflammatory biomarkers of asymptomatic and symptomatic tuberculosis

**DOI:** 10.1038/s41467-026-75909-6

**Published:** 2026-07-25

**Authors:** Denis Awany, Dominique T. Ariefdien, Simon C. Mendelsohn, Virginie Rozot, Humphrey Mulenga, Sarah Nyangu, Michele Tameris, Tumelo Moloantoa, Austin Katona, Fernanda Maruri, Firdows Noor, Ravindre Panchia, Khuthadzo Hlongwane, Kim Stanley, Yuri F. van der Heijden, Katie Hadley, Andrew- Fiore Gartland, Craig Innes, William Brumskine, Keertan Dheda, Shameem Jaumdally, Tahlia Perumal, Neil Martinson, Al Leslie, Bernard Fourie, Andriëtte Hiemstra, Stephanus T. Malherbe, Gerhard Walzl, Kogieleum Naidoo, Gavin Churchyard, Novel N. Chegou, Timothy R. Sterling, Mark Hatherill, Thomas J. Scriba

**Affiliations:** 1 South African Tuberculosis Vaccine Initiative, Institute of Infectious Disease and Molecular Medicine and Division of Immunology, Department of Pathology, University of Cape Town, Cape Town, South Africa; 2 Perinatal HIV Research Unit, University of the Witwatersrand, Johannesburg, South Africa; 3 Vanderbilt Tuberculosis Center, Vanderbilt University Medical Center, Nashville, TN USA; 4 South African Medical Research Council Centre for TB Research; Division of Immunology, Department of Biomedical Sciences, Faculty of Medicine and Health Sciences, Stellenbosch University, Cape Town, South Africa; 5 The Aurum Institute, Johannesburg, South Africa; 6 Vaccine and Infectious Disease Division, Fred Hutchinson Cancer Research Center, Seattle, WA USA; 7 Centre for Lung Infection and Immunity, Division of Pulmonology, Department of Medicine and UCT Lung Institute and South African MRC/UCT Centre for the Study of Antimicrobial Resistance, University of Cape Town, Cape Town, South Africa; 8 Faculty of Infectious and Tropical Diseases, Department of Immunology and Infection, London School of Hygiene and Tropical Medicine, London, UK; 9 Africa Health Research Institute, Durban, South Africa; 10 Division of Infection and Immunity, University College London, London, UK; 11 Department of Medical Microbiology, University of Pretoria Faculty of Health Sciences, Pretoria, South Africa; 12 Centre for the AIDS Programme of Research in South Africa (CAPRISA), Durban, South Africa; 13 MRC-CAPRISA HIV-TB Pathogenesis and Treatment Research Unit, Doris Duke Medical Research Institute, University of KwaZulu-Natal, Durban, South Africa; 14 School of Public Health, University of the Witwatersrand, Johannesburg, South Africa; 15 Department of Paediatrics and Child Health, University of Cape Town, Cape Town, South Africa; 16 Colorado State University, Fort Collins, USA; 17 University of Washington, Seattle, USA; 18 University of California San Francisco, San Francisco, USA

**Keywords:** Diagnostic markers, Tuberculosis, Chronic inflammation

## Abstract

A large proportion of individuals with tuberculosis (TB) are asymptomatic. The biological and inflammatory underpinnings of asymptomatic TB are unknown and may differ from symptomatic TB. We characterise blood transcriptomic and proteomic profiles in South African community screening vs. health facility-based triage cohorts. Asymptomatic TB shares core transcriptomic and proteomic features with symptomatic TB, including upregulation of innate, interferon and inflammatory pathways and downregulation of T and B cell pathways. Integration of transcriptomic and proteomic data from asymptomatic TB individuals identifies two distinct sub-clusters characterized by higher or lower bacterial burden, blood IFN-γ responses, BMI, and chest radiographic abnormalities, suggesting different disease severity. We identify a new blood transcriptomic signature of asymptomatic TB. However, diagnostic performance of transcriptomic and proteomic markers is weaker for asymptomatic TB than symptomatic TB, suggesting that policy development for community-based, asymptomatic TB screening should not adopt biomarkers developed for symptomatic TB triage without further optimization.

## Abstract (web summary)

A large number of individuals with tuberculosis (TB) are asymptomatic. Here, the authors characterise blood transcriptomic and proteomic profiles in South African community screening cohorts to identify inflammatory signatures of asymptomatic TB.

## Introduction

Morethan halfof all individuals with tuberculosis (TB) identified in national TB prevalence surveys did not have, recognize, or report typical TB symptoms such as cough, fever, night sweats, and loss of weight^1^, and were classified as asymptomatic^2,3^. Three studies in South Africa that performed universal community or household contact TB screening, irrespective of symptoms or chest radiographic findings, reported even higher proportions of asymptomatic TB, above 80%^4–6^, while a study in high-risk groups found that 55% of clinic attendees with bacteriologically-confirmed TB were asymptomatic^7^. Recent studies also suggest that symptoms, such as cough, are not required for aerosol release of *M. tuberculosis*^8,9^, raising the possibility that asymptomatic TB may contribute to transmission. Indeed, modeled projections based on data from 25 historic cohorts suggest that 52% of *M. tuberculosis* transmission was from individuals with asymptomatic disease^10,11^. Thus, asymptomatic TB is not only common, but finding, treating and preventing asymptomatic TB may be important for the success or failure of global TB control measures.

Yet, a number of fundamental questions remain unanswered, including whether asymptomatic TB differs from symptomatic disease in its biology, severity, morbidity, risk of post-TB lung disease or mortality, and likelihood of detection by host biomarkers and molecular diagnostic tests. A critical unknown for global health policy-makers is how diagnostic tools originally developed for health facility-based triage of persons presenting with symptoms suggestive of TB to primary health clinics will perform for community-based screening of asymptomatic TB. A major obstacle is that those with asymptomatic TB do not self-present for care and are not detected by active symptom screening in facilities or communities. As a result, asymptomatic TB is poorly understood, and the host immune and inflammatory responses in asymptomatic TB remain to be characterized. We recently assessed several published blood transcriptomic signatures of TB with promise as tests for TB triage^12^, predicting progression to TB disease^13–16^, and diagnosis, in the CORTIS studies. We observed promising diagnostic performance among symptomatic participants for many signatures, but specificity for asymptomatic TB was sub-optimal. At 90% sensitivity, the specificity for all signatures fell below 50%^17^, which falls well short of the minimum criteria set out in the Target Product Profile (TPP) for a TB screening test^18^. New symptom-agnostic tools are needed for community-based TB screening. However, if asymptomatic TB in the community differs in underlying mechanism and/or severity from symptomatic TB detected at health facilities, different screening approaches may be needed.

Radiological imaging in close contacts of TB cases suggest a large range of pulmonary and other thoracic manifestations and ^18^F-Fluorodeoxyglucose positron emission and computed tomography (PET/CT) imaging identifies a high degree of variability in inflammatory and metabolic activity^19^. Importantly, individuals with PET-CT-active lesions were at significantly higher risk of subsequent progression to bacteriologically-positive TB than those with PET-CT-inactive lesions^19^. Studies that screen for asymptomatic TB among contacts, thus, may include individuals with early disease, who are sampled while progressing from *M. tuberculosis* infection to symptomatic TB, while others have undulating disease that cycles through asymptomatic and symptomatic stages^20^. Like symptomatic TB^21^, asymptomatic TB may encompass a spectrum of disease states with distinct biological and inflammatory responses.

Here, we characterized the inflammatory response in individuals with bacteriologically-confirmed, asymptomatic TB using blood transcriptomic and proteomic profiling, and compared these profiles to symptomatic TB in contemporaneous cohorts. We also sought to identify a blood transcriptomic signature of asymptomatic TB. We show that asymptomatic TB is characterized by increases in inflammatory responses and innate immune activation previously described for symptomatic TB. However, these perturbations are less pronounced in asymptomatic TB than symptomatic TB, which may have profound implications for the success of new diagnostic tools. In addition, asymptomatic TB is a highly heterogenous, intermediate phenotype in the TB spectrum comprised of at least two subclusters, rather than a distinct phenotype. The findings suggest that host biomarkers and molecular tests developed and validated for facility-based triage of symptomatic TB cannot simply be repurposed for community-based screening of asymptomatic TB without further optimization.

## Results

### Participant characteristics

**Fig. 1:**
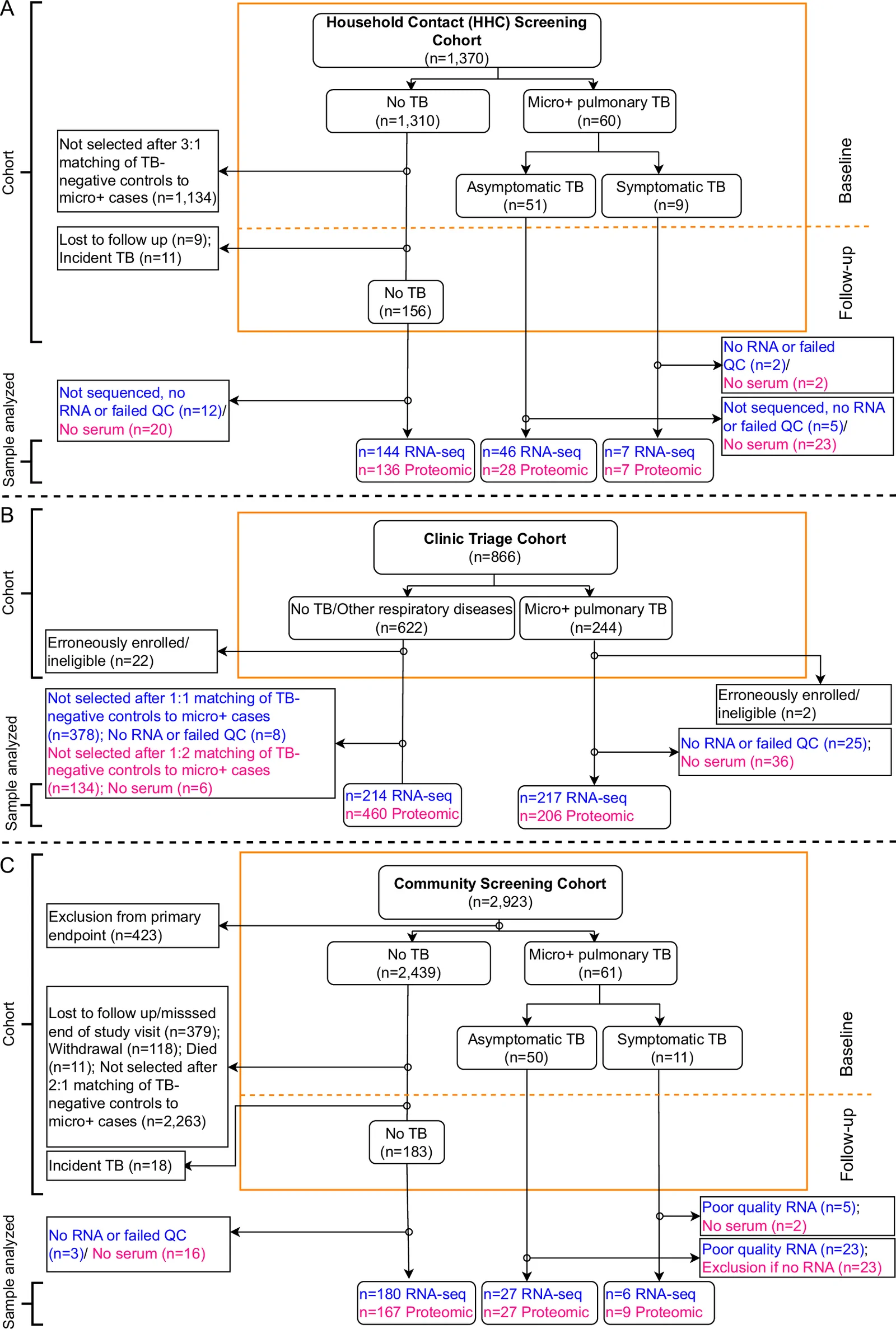
Participant and sample selection for the transcriptomic and proteomic analyses in the three cohorts. The diagrams illustrate enrollment, classification of TB cases and controls and inclusion and exclusion of samples for transcriptomic (blue) or proteomic (red) analyses for the **A** Household contact (HHC) screening cohort, **B** clinic triage cohort, and **C** community screening cohort. For each cohort, stratification into asymptomatic TB, symptomatic TB, and TB-negative (no TB) is shown. Subsequent exclusions for reasons such as loss to follow-up, poor sample quality (“failed QC”), and case-control selection are indicated, leading to the final number of samples analysed for RNA-seq and proteomics for each group.

Among HHC Screening Cohort participants, RNA-seq data for blood transcriptomic profiling was available for 46 asymptomatic TB cases, 7 symptomatic TB cases, and 144 controls (Fig. 1A). Among Clinic Triage Cohort participants, RNA-seq data were available for 217 symptomatic care-seeking TB cases and 214 controls without TB, who likely had other respiratory diseases (ORD, Fig. 1B). Among community screening cohort participants, RNA-seq data were available for 27 asymptomatic TB cases, 6 symptomatic TB cases, and 180 controls (Fig. 1C). The demographic characteristics of the participants are summarized in Supplementary Fig. 1 and Supplementary Table 1.

### Transcriptomic profiles of asymptomatic TB

**Fig. 2:**
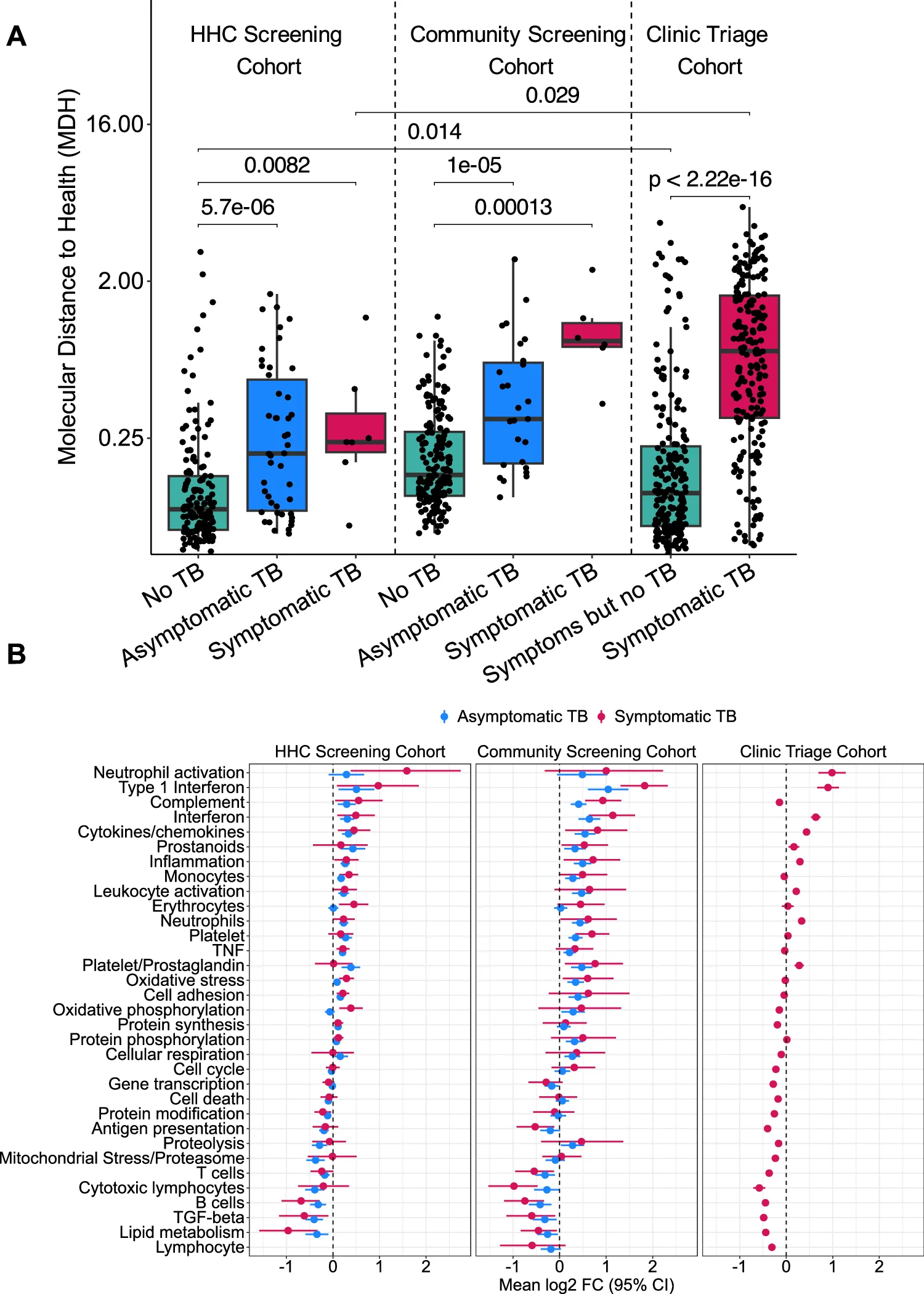
Blood transcriptome perturbation and pathways, biological processes, and gene sets modulated in symptomatic and asymptomatic TB. **A** Box and whisker plots showing molecular distance to health, a composite score that quantifies transcriptome-wide deviation from healthy controls, integrating both the number and magnitude of significantly regulated gene transcripts. On the left are Household Contact (HHC) Screening participants without TB (*n* = 144), with asymptomatic TB (*n* = 46) or symptomatic TB (*n* = 7). In the middle are community screening participants without TB (*n* = 180), with asymptomatic TB (*n* = 27) or symptomatic TB (*n* = 6). On the right are Clinic Triage participants without TB (*n* = 212) or bacteriologically-confirmed symptomatic TB (*n* = 197). The horizontal lines indicate the median, the boxes the interquartile range (IQR) and the whiskers the range of data with 1.5×IQR from the lower and upper quartiles. The shown *p* value was computed with a two-sided Mann–Whitney *U*-test. **B** Differential gene set enrichment analysis using QuSAGE on blood RNA-sequencing data, for the gene sets/modules listed on the left, from individuals with asymptomatic TB or symptomatic TB, relative to TB-negative controls, in the HHC screening cohort (left; *n* = 144, no TB; *n* = 46, aTB; *n* = 7, sTB), community screening cohort (middle; *n* = 180, no TB; *n* = 27, aTB; *n* = 6, sTB), and clinic triage cohort (*n* = 212, no TB; *n* = 197, sTB). Dots denote the mean enrichment score estimate and error bars the 95% confidence interval. The order of the gene sets is based on ranking from highest to lowest enrichment score estimates in prevalent TB (symptomatic and asymptomatic combined) from the HHC screening cohort.

We first computed the molecular distance to health (MDH)^22^, a composite score of the number of transcripts in the transcriptome that significantly differ from healthy controls and the degree of the difference in expression levels. Symptomatic TB cases who presented to health facilities (clinic triage) had markedly higher MDH scores than those without TB, who had ORD (Fig. 2A). Strikingly, although individuals with asymptomatic TB in both Screening cohorts had significantly higher MDH scores than TB-negative controls, these scores were much lower than in symptomatic TB in the Clinic Triage Cohort (Fig. 2A). This suggests that asymptomatic TB may be characterized by less perturbation of blood leukocyte gene expression and lower inflammatory responses and suggests that those with asymptomatic TB may have less severe disease. The absolute number of differentially expressed genes also supported this pattern (Supplementary Fig. 2). To assess this hypothesis further, we compared microbiological features between these groups. Symptomatic TB cases among the Clinic Triage Cohort also had lower BMI, and lower time to culture positivity than asymptomatic TB cases (Supplementary Fig. 2), further supporting that those with asymptomatic TB may have less severe disease.

To explore pathways and processes of the transcriptional host response during asymptomatic TB, we performed gene set enrichment analysis (GSEA) using blood transcriptional modules (BTM)^23^, contrasting asymptomatic TB transcriptomes with those from TB-negative controls (Fig. 2B) among the HHC screening and community screening cohorts. In both these cohorts, asymptomatic TB was associated with significantly elevated gene modules that reflect innate, inflammatory, and myeloid processes, including interferon (IFN) and type I IFN, cytokines and chemokines, inflammation, complement activation, neutrophil activation, leukocyte activation, monocytes, prostanoids, platelet/prostaglandin, and TNF. Cellular processes and metabolism pathways, such as oxidative stress, protein phosphorylation, oxidative phosphorylation, and cellular respiration, were also elevated in asymptomatic TB, relative to TB-negative controls. By contrast, lymphoid pathways, including B cells, T cells, cytotoxic T lymphocytes, lipid metabolism, and TGFβ pathways were significantly lower in asymptomatic TB compared with controls (Fig. 2B). Among these two screening cohorts similar perturbations of pathways were observed in symptomatic TB, although these results were more variable than observed in asymptomatic TB, likely due to the much smaller sample sizes of symptomatic TB in the HHC and community screening cohorts. Broadly similar patterns were observed when symptomatic TB detected among Clinic Triage participants was contrasted with TB-negative ORD controls, as extensively reported when symptomatic TB has been compared to TB-negative controls^22,24–26^. However, it was notable that complement activation, monocytes, platelets, TNF, and cellular processes and metabolism pathways were not significantly elevated. This is likely because symptomatic TB cases were compared to symptomatic, care-seeking controls with other respiratory diseases, rather than healthy controls, which have been a more commonly used comparator group for gene module and pathway analyses in published studies.

In light of the consistent patterns observed in the HHC Screening and Community Screening cohorts, and to provide a larger sample size to explore cellular and inflammatory processes across the TB disease spectrum, we combined RNA-seq data from these cohorts and performed weighted gene co-expression network analysis (WGCNA), a clustering method that reduces high dimensional data, such as full transcriptomes, into modules that preserve intrinsic relationships between variables within a network structure^27,28^.

**Fig. 3:**
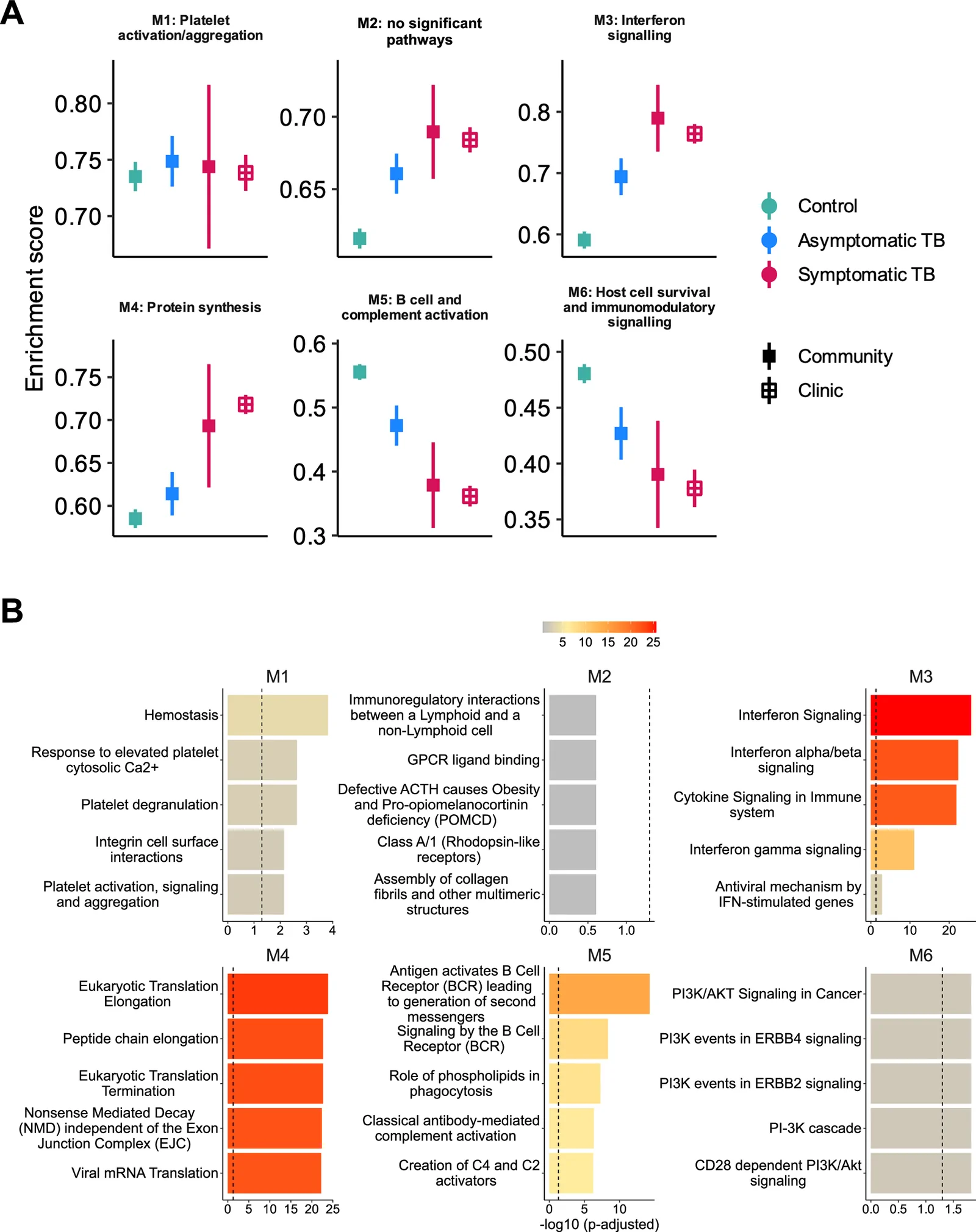
Blood transcriptome perturbations increase progressively along the TB spectrum. Analysis of RNA-seq data from individuals categorized and ranked along the TB spectrum, from healthy controls (*n* = 523), asymptomatic TB (*n* = 73), symptomatic TB (*n* = 210) in community screening participants and symptomatic TB in clinic triage participants, by weighted gene co-expression network analysis using CEMiTool. For these analyses, data from the HHC Screening (RePORT SA) and Community Screening (CORTIS) cohorts were combined. **A** M1 through M6 represent six modules of co-expressed gene networks identified by CEMiTool. Dots denote the mean enrichment score estimate and error bars the 95% confidence interval. **B** Gene set overrepresentation analysis for the identified gene modules. Displayed are the top five significantly enriched pathways and processes for each of the six modules, ranked within each module according to Benjamini–Hochberg-adjusted *p* value. The color intensity for each module corresponds to the magnitude of the FDR *p* value. Expression levels of representative top regulated genes within each module and pathway along the TB spectrum is shown in Supplementary Fig. 3.

WGCNA using the top 75% most variable genes identified six major modules for which enrichment scores could be computed (Fig. 3A). When aligned along the TB spectrum, spanning no-TB controls, asymptomatic TB, and symptomatic TB, which were further stratified into Community Screening and Clinic Triage cohorts, enrichment scores for M2 (no significant pathways identified), M3 (interferon signaling), and M4 (protein synthesis) increased progressively along this trajectory, while enrichment scores for M5 (B cell and complement activation) and M6 (host cell survival and immunomodulatory signaling) decreased progressively (Fig. 3A, B).

M3 showed highly significant enrichment for innate inflammatory pathways well known to be upregulated in TB^22,26,29^, including IFN, type I IFN, and IFN-γ signaling and cytokine signaling (Fig. 3B). Expression of several canonical IFN-stimulated genes, which are common components of transcriptomic signatures of TB, including *FCGR1A*, *GBP1*, *GBP4* and *GBP5*, *IFIT3*, and *IFITM3* reflected this progressive increase along the TB spectrum trajectory (Supplementary Fig. 3A).

M5 showed highly significant enrichment for B cell and B cell receptor signaling pathways (Fig. 3B) and several canonical B cell genes, such as *CD19*, *CD79A*, *CD79B*, and *IGHD*, reflecting a pattern of progressive decrease along the TB spectrum trajectory (Supplementary Fig. 3B).

M4 showed enrichment of pathways associated with translation and peptide chain elongation and included genes encoding ribosomal proteins, such as *RPL31*, *RPL34*, *RPL7*, and *RPS7* (Supplementary Fig. 4A).

No significant enrichment for a highly informative pathway could be identified for M2 or M6 (Fig. 3B), although progressive decreases along the TB spectrum were observed for M6 genes modulated by signal transduction through the fibroblast growth factor receptor (*FGFR*), such as *CAMK4*, *CD28*, *THEM4*, and *RASGRF2* (Supplementary Fig. 4B).

To confirm that these findings were not driven by batch-correction artifacts or cohort integration effects, WGCNA was repeated independently for each of the three cohorts (HHC screening, community screening, and clinic triage cohort), without cross-cohort integration. Consistent module-disease associations were observed for interferon signaling, protein synthesis and B/T cell signaling across the cohorts (Supplementary Fig. 13), suggesting that the identified transcriptional patterns represent robust biological signals.

### Performance of published transcriptomic TB signatures in asymptomatic TB

Next, we sought to determine how previously published, validated transcriptomic signatures of TB perform as diagnostic biomarkers of asymptomatic TB. We selected a set of signatures that were developed as diagnostic biomarkers for TB (Kaforou24^25^, Maertzdorf4^30^, Sweeney3^31,32^, XpertHR^33^), prognostic biomarkers for TB (Gliddon3^34^, Penn-Nicholson6^35^, Roe1^36^, and Suliman4^37^), a pediatric TB signature (Thakur9^38^), and a signature of *M. tuberculosis* containment in mice with promising performance in humans (Duffy5^39^). All 10 signatures significantly discriminated between asymptomatic TB and healthy controls in both the HHC Screening and the Community Screening cohorts, with largely similar AUROC values (Supplementary Fig. 6). AUROC values were generally higher (AUROCs mostly above 0.75) in the latter (AUROCs mostly below 0.75) than the former cohort. However, it was notable that discriminatory performance for all signatures was better for symptomatic TB than for asymptomatic TB, with the exception of the Duffy5 signature. Analysis of published, validated transcriptomic signatures of TB therefore showed the same pattern as reported above, namely that asymptomatic TB was characterized by less systemic inflammatory signal than symptomatic TB.

### Serum proteomic profiles of asymptomatic TB

We then explored changes in acute response, inflammatory, and immune response serum protein mediators associated with asymptomatic TB, relative to symptomatic TB. Among HHC Screening Cohort participants, serum protein data were available for 28 asymptomatic TB cases, 7 symptomatic TB cases, and 136 controls. Among symptomatic Clinic Triage Cohort participants, serum protein data were available for 206 TB cases and 460 ORD controls, and among Community Screening Cohort participants, protein data were available for 27 asymptomatic TB cases, 6 symptomatic TB cases, and 180 controls (Supplementary Table 1).

**Fig. 4:**
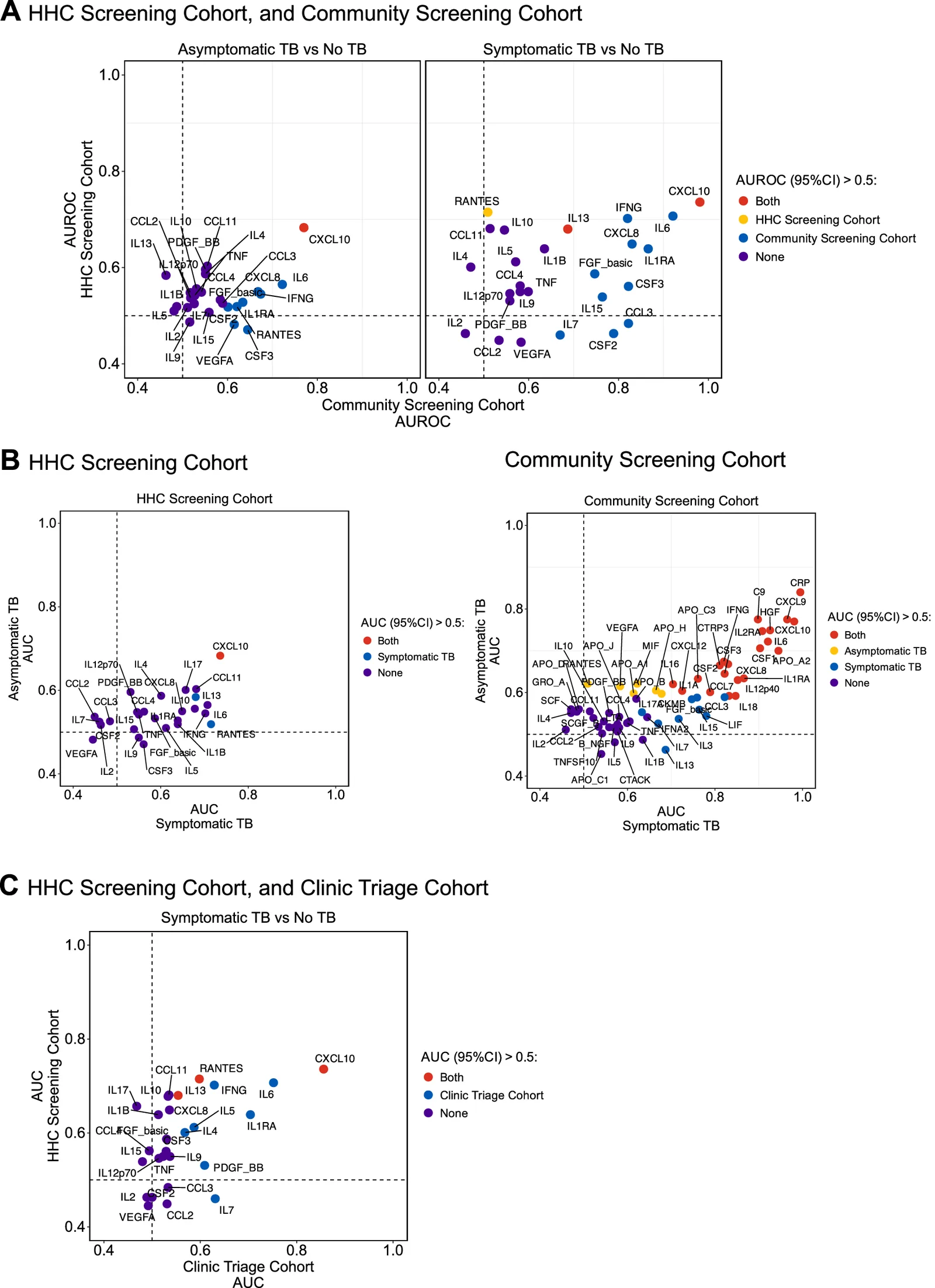
Proteomic biomarkers of asymptomatic TB. Scatter plots summarizing the diagnostic performance (AUROC) of proteomic biomarkers for distinguishing between asymptomatic TB (*n* = 28) and TB-negative controls (*n* = 136) in HHC screening (RePORT SA, Y-axis) and community screening (CORTIS, X-axis; aTB (*n* = 52), No TB (*n* = 167)) participants or between sTB (*n* = 206) and No TB controls (*n* = 460) in clinic triage participants. Each point represents a protein, with its position reflecting AUROC values for the relevant comparison. Points are color-coded according to their lower 95% confidence interval (CI) value for the AUROC; biomarkers with lower 95% CI values below 0.5 were considered to display no significant differentiation between the groups (purple), those with lower 95% CI values above 0.5 in only one cohort are in yellow or blue, and those with lower 95% CI values above 0.5 in both cohorts are in red. **A** AUROC in asymptomatic TB vs control (left) and symptomatic TB vs control (right) in the HHC screening (Y-axis), and community screening (X-axis) cohorts for proteins measured in both cohorts. **B** AUROC in symptomatic TB vs controls in HHC screening (Y-axis), and in clinic triage participants (X-axis) for protein markers measured in both cohorts. **C** AUROC in asymptomatic TB vs control (Y-axis) and symptomatic TB vs control (X-axis) in the HHC screening (left), and in community screening (right) cohorts using all proteins measured in the respective cohorts. This visualization highlights both cohort-specific and phenotype-specific performance as well as biomarkers with reproducible diagnostic potential. Data for each protein are in Supplementary Fig. 5.

Overall, diagnostic performance for distinguishing TB from TB-negative controls across community screening cohorts, measured by AUROC, was consistently higher for individuals with symptomatic TB compared with those with asymptomatic TB. This trend was observed in both HHC and community screening cohorts, indicating stronger discriminatory signal in symptomatic disease. Among the top-performing biomarkers were CXCL10, IFN-γ, and IL-6. In the HHC Screening Cohort, these markers attained AUROC 0.683 (95% CI 0.583–0.783), 0.545 (95% CI 0.427–0.663), and 0.565 (95% CI 0.460–0.670) in asymptomatic TB, and 0.74 (95% CI 0.546–0.926), 0.720 (95% CI 0.444–0.960), and 0.707 (95% CI 0.472–0.942) in symptomatic TB respectively. In the Community Screening Cohort, the discriminatory power was 0.770 (95% CI 0.695–0.846), 0.674 (95% CI 0.590–0.758), and 0.722 (95% CI 0.638–0.806) in asymptomatic TB, and 0.981 (95% CI 0.965–0.998), 0.820 (95% CI 0.641–1.00), and 0.921 (95% CI 0.879–0.962) in symptomatic TB respectively (Fig. 4A and Supplementary Fig. 7). CXCL10 consistently ranked the best in both asymptomatic and symptomatic across both cohorts. Notably, performance metrics were generally higher in the Community than the HHC screening cohort, suggesting possible cohort-specific difference in disease severity and/or host response (Fig. 4A, B and Supplementary Fig. 7A, B).

We next evaluated these proteins in the Clinic Triage Cohort. A number of proteins showed good diagnostic performance, with the top performing ones including CXCL10 (AUROC 0.856; 95% CI 0.822–0.889%), IL-6 (AUROC 0.752; 95% CI 0.713–0.791), IL-1RA (AUROC 0.704; 95% CI 0.660–0.749), IL-7 (AUROC 0.631; 95% CI 0.586–0.675), and IFN-γ (AUROC 0.629; 95% CI 0.583–0.674) (Fig. 4C and Supplementary Fig. 7C). Overall, the performance in this cohort was higher than in the asymptomatic and symptomatic cases from the HHC Screening Cohort.

### A transcriptomic signature of asymptomatic TB

**Fig. 5:**
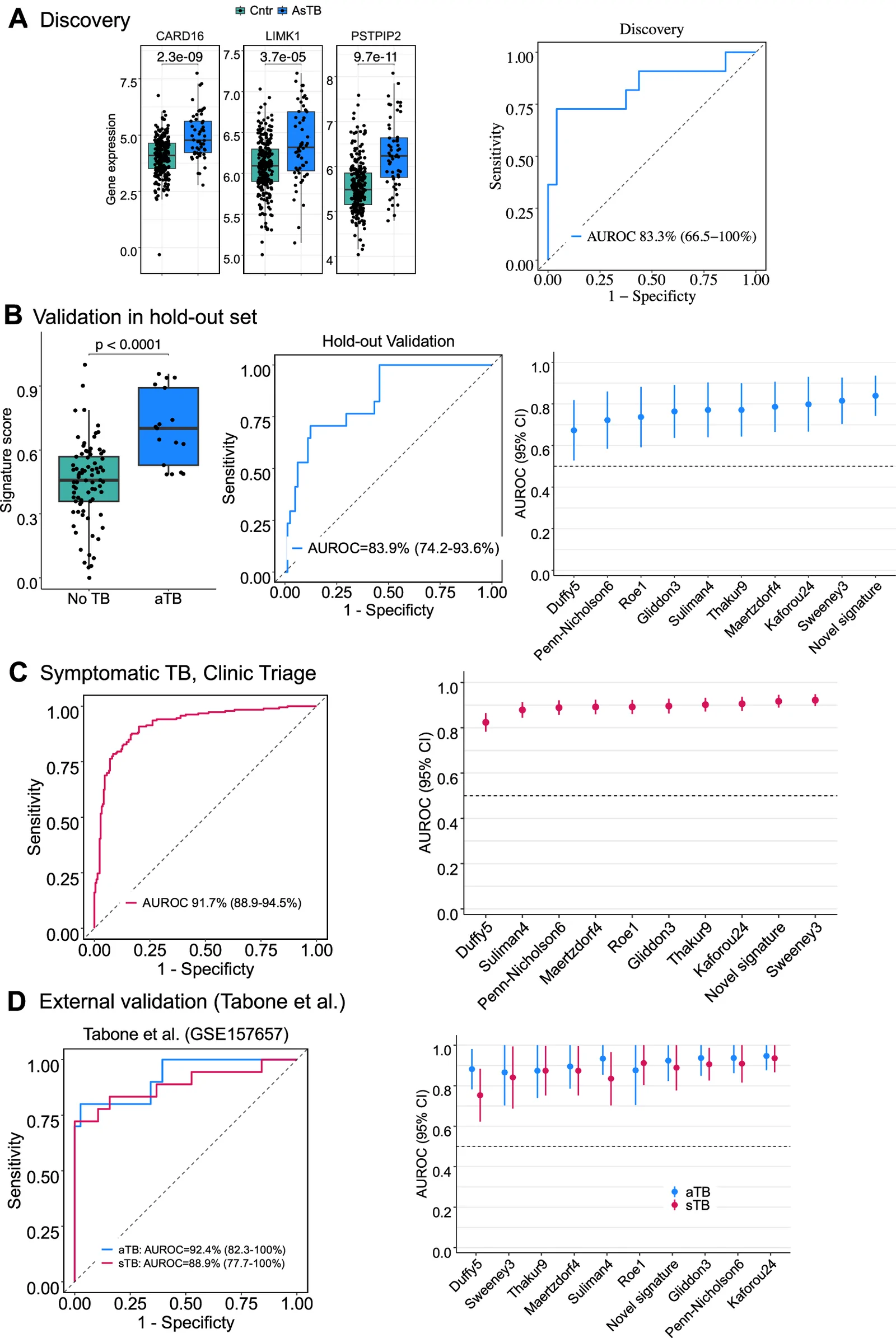
Discovery and validation of a transcriptomic signature of asymptomatic TB. Data from the HHC screening (RePORT SA) and community screening (CORTIS) cohorts were combined and partitioned into discovery (training, 75%, 21 + 35 aTB and 135 + 108 healthy controls, respectively) and hold-out validation (25%, 6 + 11 aTB cases and 45 + 36 healthy controls, respectively) sets. Feature selection model fitting into a three-gene signature of aTB, in the discovery subset. **A** Expression levels of genes in the novel asymptomatic TB signature in the discovery set and ROC curve plot for its performance (model fit) in the discovery set. *p*-values were computed with a two-sided Mann–Whitney *U*-test. **B** Asymptomatic TB signature scores in the hold-out validation set. Horizontal lines indicate medians, boxes the interquartile range (IQR) and whiskers the range of data with 1.5×IQR from the lower and upper quartiles. *p* value was computed with a two-sided Mann–Whitney *U*-test. Performance of the novel signature in the hold-out validation set for differentiating asymptomatic TB cases from healthy controls. The right-hand plot shows AUROC values (dots) and 95% CI (error bars) of the novel asymptomatic TB signature and previously published TB signatures in the validation set. Dots denote the AUC estimate and error bars the 95% confidence interval. **C** ROC curve of the novel asymptomatic TB signature for differentiating between symptomatic TB (*n* = 186) and symptomatic TB-negative controls (*n* = 214) in the Clinic Triage cohort (RePORT SA). The right-hand plot shows AUROC values (dots) and 95% CI (error bars) of the novel signature and previously published TB signatures in the clinic triage cohort. Dots denote the AUC estimate and error bars the 95% confidence interval. **D** ROC curves of the novel asymptomatic TB signature for differentiating between asymptomatic TB cases (*n* = 10, blue) or symptomatic TB cases (*n* = 18, red) and controls (*n* = 38) in datasets (GSE157657) from Tabone et al., 2017. The right-hand plot shows AUROC values (dots) and 95% CI (error bars) of the novel asymptomatic TB signature and previously published TB signatures in the GSE157657 dataset. Dots denote the AUC estimate and error bars the 95% confidence interval.

Given that previously discovered transcriptomic biomarkers performed poorer for asymptomatic than symptomatic TB, we reasoned that a distinct host response in asymptomatic TB may require a signature that is specific to this manifestation within the TB spectrum. We therefore aimed to discover a signature of asymptomatic TB. To overcome differences in design between the HHC Screening (RePORT SA) and Community Screening (CORTIS) cohorts, we combined these cohorts and then partitioned them into discovery (training, 75%) and validation (25%) sets (Fig. 5). Feature selection and model fitting in the discovery subset resulted in a novel 3-gene signature of asymptomatic TB, comprising *PSTPIP2*, *CARD16*, and *LIMK1*, which discriminated between asymptomatic TB and healthy controls with an AUROC of 83.3% (95% CI 66.5–100%) (Fig. 5A).

When applied to the held-out validation set, scores for the novel signature were significantly higher in asymptomatic TB than TB-negative controls; and discriminated between these groups with an AUROC of 83.9% (95% CI 74.2–93.6%, Fig. 5B). The novel signature nominally had the highest AUROC, but was not significantly better than any of the other ten transcriptomic signatures. When we analysed diagnostic performance for TB among symptomatic Clinic Triage Cohort participants, AUROC for the novel signature was 91.9% (95% CI 89.2–94.7%), which was also not different from nine of the ten published signatures (Fig. 5C). Finally, we also applied these signatures to a publicly available dataset from HHC in Leicester, UK, described by ref. ^40^, which comprised 38 healthy controls, seven individuals (ten samples) with “subclinical TB”, defined as having no symptoms but with radiological changes or microbiological evidence of active TB, and 14 individuals with symptomatic, “clinical TB” (microbiologically confirmed). The novel signature, along with nine of the ten other signatures, discriminated between controls and “subclinical TB” or “clinical TB” with similar performance, as reported for multiple signatures in ref. ^40^ (Fig. 5D). Taken together, these results suggest that the performance of our novel asymptomatic TB signature was not significantly different to published signatures that were trained for symptomatic TB, progression to incident TB, or TB treatment monitoring.

### Identifying asymptomatic TB subtypes

**Fig. 6:**
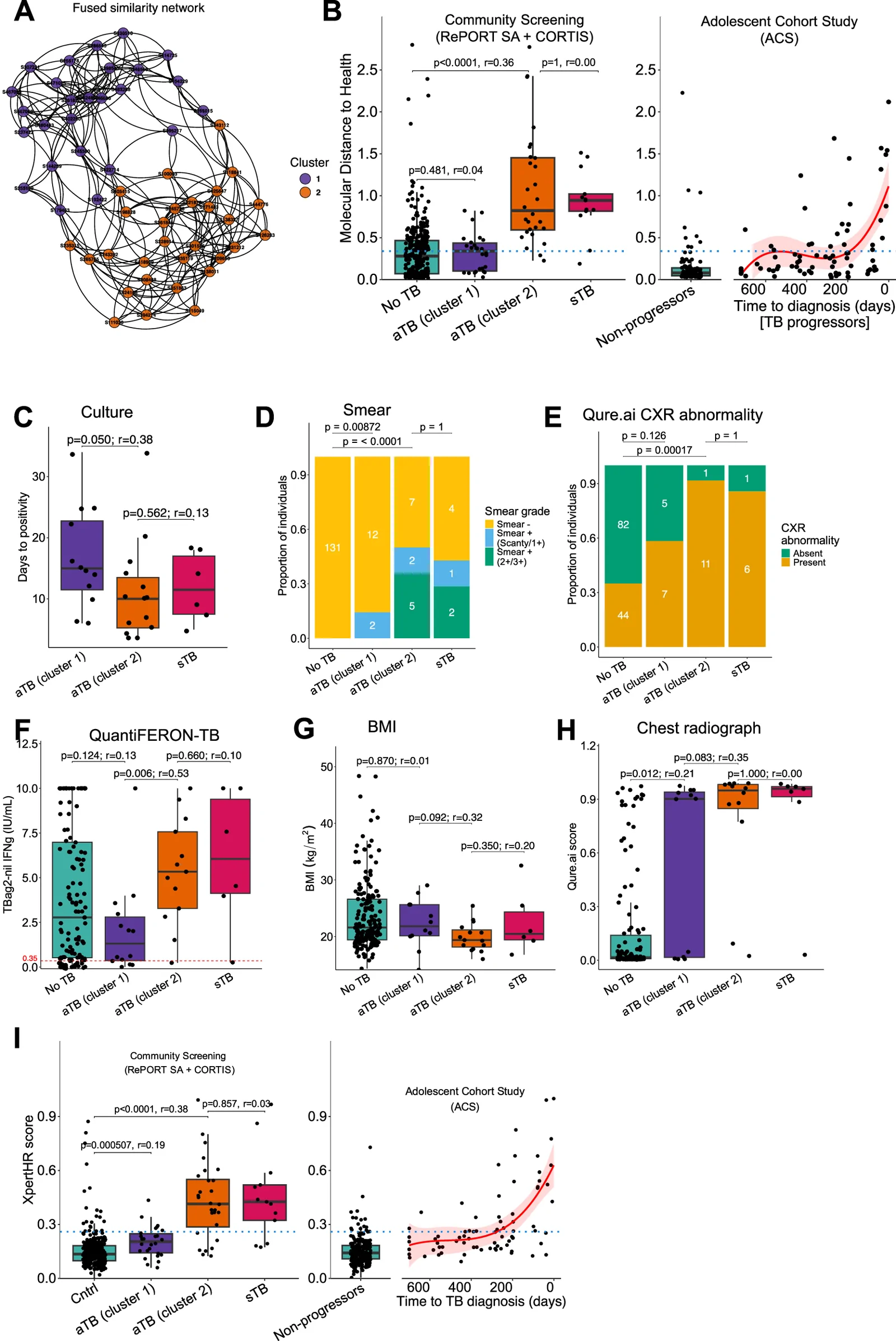
Two asymptomatic TB clusters based on transcriptomic and proteomic data. **A** Similarity network fusion (SNF) analysis on the HHC and community screening cohorts combined identified two aTB clusters (cluster 1, *n* = 26; cluster 2, *n* = 28). **B** MDH from HHC Screening cohort (sTB, *n* = 7; aTB, *n* = 27; no TB, *n* = 141), the community screening cohort (*n* = 180, No TB; 26, aTB cluster 1; 28, aTB cluster 2; 13, sTB) and TB progressors (*n* = 76) and non-progressors (*n* = 99) from the adolescent cohort study (ACS). Time points in ACS progressors were aligned to time-t-diagnosis and a spline fitted (shaded area, 95% CI). **C** Sputum bacteriological outcomes, including days to culture positivity (those with culture-positive results; *n* = 12, aTB cluster 1; *n* = 14 aTB cluster 2; *n* = 6 sTB), **D** sputum smear microscopy grade (*n* = 131 No TB; *n* = 14 aTB cluster 1; *n* = 14 aTB cluster 2; *n* = 7 sTB), and **E** CXR abnormality by Qure.ai analysis in asymptomatic TB clusters 1 (*n* = 12) and 2 (*n* = 12) and participants with sTB (sn = 7) or without TB (*n* = 126) in the HHC and community screening cohorts. **F** IFN-γ levels from QuantiFERON assays (*n* = 125 No TB; *n* = 14, aTB cluster 1; *n* = 13, aTB cluster 2; *n* = 6, sTB), **G** BMI values (*n* = 180, No TB; *n* = 12, aTB cluster 1; *n* = 15, aTB cluster 2; *n* = 6, sTB), and **H** Qure.ai CXR scores (*n* = 126, No TB; *n* = 12, aTB cluster 1; *n* = 12, aTB cluster 2; *n* = 7, sTB). **I** XpertHR signature score in HHC screening and community screening combined, and ACS progressors (*n* = 76) and non-progressors (*n* = 99). The non-linear spline was fitted to MDH scores (shaded area indicates 95% CI). BMI was only available for CORTIS; analysis of smear grade, QuantiFERON Gold-Plus results, and CXR scores in RePORT SA only; culture was available for both cohorts. The shown *p*-values in (**B**, **C**, **F**, **G**, **H**, **I**) were computed with a two-sided Mann–Whitney *U*-test. For (**D**, **E**) shown *p* values were computed using the Fisher exact test. For box-and-whisker plots, horizontal lines indicate medians, boxes the IQR and whiskers the range of data with 1.5×IQR from the lower and upper quartiles.

A critical question is whether there is clustering of features among asymptomatic TB individuals that supports the existence of subtypes that may be associated with clinically relevant factors, such as disease severity or infectiousness. Similarity network fusion (SNF) analysis of the full transcriptomic and proteomic datasets from individuals with asymptomatic TB, combined from the two community screening cohorts, revealed two clusters (Fig. 6A) by both the rotation cost and Eigen-gap methods. The fully fused network exhibited significant internal stability (ADM = 0.03, and APN = 0.07; Supplementary Fig. 8) and median ARI was 90.9% (IQR 82.23–90.91%) across 500 resampling iterations, each involving a random selection of 80% of the participant samples, in line with established methodological practices^41^. Interestingly, individuals in asymptomatic cluster 1 had low MDH scores at levels observed in controls without TB, while those in asymptomatic cluster 2 had significantly higher MDH scores, at levels observed in symptomatic TB (Fig. 6B). These results suggest that asymptomatic individuals in TB cluster 2 may have more systemic inflammation than those in cluster 1.

To explore whether disease severity differed between the two asymptomatic TB clusters, we compared sputum microbiological outcomes. Individuals in cluster 2 had a lower time to sputum MGIT culture positivity (Fig. 6C) and a higher proportion of 2+ or 3+ sputum smear grade (Fig. 6D), all consistent with higher sputum bacterial burden, than individuals in cluster 1. More individuals in cluster 2 were classified as having CXR abnormalities (Fig. 6E), while these individuals also had significantly higher levels of QFT Plus IFN-γ levels (Fig. 6F), and trends to lower BMIs (Fig. 6G) and higher Qure.ai CXR scores, than individuals in cluster 1 (Fig. 6H), further supporting that those in cluster 2 had more severe disease than those in cluster 1. These differences were not confounded by key covariates such as age, sex, BMI or recruitment-related factors, supporting the interpretation that the observed transcriptional differences reflect biologically meaningful variation rather than technical or cohort-related effects (Supplementary Table 2).

Finally, we compared XpertHR transcriptomic TB signature scores in the two asymptomatic TB clusters. As observed for MDH (Fig. 6B), XpertHR signature scores were significantly higher in cluster 2 individuals than those in cluster 1 (Fig. 6I), suggesting higher systemic inflammatory responses in cluster 2 that were at levels observed in symptomatic TB. Consistent with this, cluster 1 had similar Penn-Nicholson6^35^ and Duffy5^39^ transcriptomic signature scores to No-TB controls, while cluster 2 had similar signature scores to symptomatic TB cases (Supplementary Fig. 9). The same was observed for granulocyte/lymphocyte ratio (Supplementary Fig. 9), also a marker of systemic inflammation computed by transcriptome deconvolution. To further contextualize these asymptomatic TB transcriptional changes within the continuum of TB progression, we analysed whole blood RNA-seq data from M.tb-infected TB progressors and non-progressors from the longitudinal Adolescent Cohort Study (ACS)^29,42^. MDH and XpertHR transcriptomic signature scores in TB progressors increased progressively as TB diagnosis approached, indicating gradual molecular perturbation during disease progression, reflecting systemic inflammation. MDH and XpertHR scores at levels observed in asymptomatic TB cluster 2, who had sputum bacterial burden and CXR abnormalities akin to individuals with symptomatic TB, occurred as early as 300–200 days before symptom-triggered TB diagnosis in adolescent progressors. MDH and XpertHR scores in progressor samples collected more than a year before TB diagnosis were typically much lower, at levels observed in individuals in asymptomatic TB cluster 1 (Fig. 6B, I and Supplementary Methods).

## Discussion

We characterized blood transcriptomic and proteomic profiles in asymptomatic TB detected by household and community screening, compared to symptomatic TB detected by facility-based triage, in contemporaneous cohorts using standardized approaches. Our results show that systemic inflammatory profiles among persons with asymptomatic TB, including proteomic and transcriptomic signatures and MDH were highly heterogenous; and of significantly lower magnitude than inflammatory profiles among individuals with symptomatic TB, whether detected in the community or presenting to clinics. These findings are consistent with previous findings from RePORT SA, showing that CRP levels, yield of CXR screening, and sputum bacillary load were significantly lower in asymptomatic TB than is typically observed in symptomatic TB^6^.

Most TB diagnostic studies enroll symptomatic patients who seek care at health facilities, typically with more advanced disease than is identified in community screening studies. Therefore, the poor performance of host response biomarkers for asymptomatic TB, relative to symptomatic TB, has important implications for the feasibility of biomarker-based screening approaches to identify individuals with undiagnosed TB in community settings. These data address two fundamental questions that may be critical for TB control: first, whether asymptomatic differs from symptomatic TB in biology and severity (it does); and second, whether biomarkers developed and validated for symptomatic TB in triage settings perform adequately for the detection of asymptomatic TB in community settings (they do not). The latter question is highly relevant, since the World Health Organization (WHO) recently combined triage and screening test guidelines under a single target product profile^18^. Yet, our findings suggest that biomarkers developed for facility-based triage of symptomatic TB cannot simply be repurposed for community-based screening of asymptomatic TB without further optimization or acceptance of lower performance^6^.

Application of published transcriptomic signatures of TB disease performed sub-optimally to distinguish between asymptomatic TB and healthy controls. We therefore set out to discover a novel, parsimonious transcriptomic signature of asymptomatic TB. This signature did not significantly outperform published transcriptomic signatures of TB in a hold-out validation cohort, nor in a previously published cohort of asymptomatic TB^40^.

GSEA and WGCNA of RNA-seq data suggested that similar biological pathways and processes were perturbed in asymptomatic TB, albeit to a lesser degree than the well-described perturbations in symptomatic TB^22,26,29,35,40,43,44^. The most prominent pathways found to be induced during asymptomatic TB were IFN responses, complement activation, inflammation, and myeloid cell activation, with peripheral depletion of lymphoid cells such as B cells, T cells, and cytotoxic lymphocytes, including NK cells. These pathways are consistent with those reported for symptomatic TB, as well as studies of TB progression, and thus our findings do not suggest that asymptomatic TB is a distinct phenotype of TB. Rather, these findings, along with MDH, WGCNA, pathway analyses and proxy measures of sputum bacillary load, indicate that asymptomatic TB occupies an intermediate state within the TB spectrum, between early disease (seen in those who ultimately progress to incident TB) and those with more severe, symptomatic disease^29,35,37,42,43^.

A major finding of our study is that clustering analysis of transcriptomic and proteomic data from individuals with asymptomatic TB segregated into two stable clusters that may represent subtypes. These two clusters differed in sputum bacillary burden, CXR extent of disease, IGRA responses, and MDH and transcriptomic signature scores, consistent with differences in extent or severity of asymptomatic disease and systemic inflammation. Important questions include whether those with more severe pulmonary disease are more likely to progress to symptomatic TB, or if they may be more infectious. Our comparisons of systemic inflammation levels in the two asymptomatic TB clusters with the longitudinal trajectories in adolescent TB progressors are suggestive of asymptomatic TB in cluster 1 temporally preceding asymptomatic TB in cluster 2, and that systemic inflammation levels observed in cluster 2 typically increase 300–200 days before TB diagnosis. Interestingly, since all individuals with asymptomatic TB in our study had microbiologically positive sputum tests, and the vast majority in asymptomatic TB cluster 2 were sputum smear-positive, our results are also consistent with respiratory bacterial shedding months or even years before TB diagnosis. Recent studies showed that symptoms, such as coughing, are not required for aerosol release of *M. tuberculosis*^8,9^, implying that individuals with asymptomatic TB may contribute to transmission of the pathogen.

Our finding of marked heterogeneity among those with asymptomatic TB is consistent with a PET-CT imaging study in close contacts of TB cases with rifampicin-resistant TB, which also showed a large range of pulmonary and other thoracic manifestations^19^. A major finding was a clear distinction between individuals with PET/CT-active and PET/CT-inactive pulmonary lesions, where the presence of PET/CT-active lesions was associated with more than 20-fold increased risk of progressing to TB disease and self-presentation with symptoms to the health services. It is possible that the two clusters we identified represent two subtypes with relatively metabolically active vs quiescent pulmonary or lymphatic lesions. A number of longitudinal studies that compared TB progressors with non-progressors have shown higher transcriptomic and proteomic signature scores, as well as activation of *M. tuberculosis*-specific T cells among progressors^4–6,17,29,34,35,37,42,43,45^. We speculate that these subtypes of asymptomatic TB might reflect pre-symptomatic progressors and others with stable, chronic, or even regressing pathology.

Future studies with longitudinal follow-up of those with asymptomatic TB are necessary to discern between those who may progress to symptomatic TB and those who may remain asymptomatic, or those who may regress to clear mycobacteria from their respiratory tract. Such studies should include detection of *M. tuberculosis*-containing bioaerosol release to inform the infectiousness of asymptomatic disease^8,9^. Such a study raises an ethical dilemma about whether people with bacterially-confirmed asymptomatic TB should receive multidrug treatment, even if they appear well and are reluctant to partake in a 4–6 month regimen.

Our study has several limitations. Although the HHC and Community Screening Cohorts were large, the yield of asymptomatic TB cases was relatively low. We cannot rule out that we have under-ascertained characteristics of asymptomatic TB; and that more subtypes might exist. We found that transcriptomic and proteomic differences between asymptomatic TB cases and controls were of a higher magnitude in the CORTIS, compared to the RePORT SA cohorts, suggesting either laboratory or assay differences, or differences in study design, study population, duration of disease, and/or time since exposure, which may be relevant to HHC cohorts. However, procedures and assays were performed in the same SATVI laboratory; and sample processing was highly consistent between the CORTIS and RePORT SA studies, ruling out technical differences as a major factor. The most likely explanation is that the CORTIS study, which pre-screened the study population to enrich for RISK11 signature-positive individuals, selected for those with more systemic inflammation. However, this factor would tend to minimize, rather than exaggerate, the differences between asymptomatic and symptomatic TB observed in this study.

Regardless, to our knowledge, this is the largest study of host blood proteomic and transcriptomic biomarkers in asymptomatic TB; and standardized data collection in contemporaneous cohorts allows direct comparison with symptomatic TB. Our findings suggest that biomarker approaches based on blood inflammatory markers are unlikely to be effective for community screening for asymptomatic TB without further optimization. Further, the clear differences in inflammation and severity of disease between individuals with symptomatic TB presenting to health facilities and those with asymptomatic TB detected in the community suggest that differences in biology should be taken into account when developing policy for TB screening and triage tests^18^.

## Methods

### Ethics statement

The research in this paper complies with all relevant ethical regulations. The RePORT SA and CORTIS study protocols were approved by the institutional research ethics committees at each participating site, including the University of Cape Town; the RePORT SA protocol was also approved at Vanderbilt University Medical Center, USA. All participants provided written informed consent prior to participation. The CORTIS clinical trial was prospectively registered at ClinicalTrials.gov under the identifier NCT02735590.

### Study design and participants

#### Regional prospective observational research for tuberculosis (RePORT) South Africa cohorts

The Regional Prospective Observational Research for Tuberculosis (RePORT) South Africa (SA) network enrolled two parallel cohorts to study novel diagnostic biomarkers, using a single standardized protocol, at six clinical sites in districts with high TB incidence rates. The first, community-based screening cohort (Fig. 1A), which includes adult (≥18 years) household contacts (HHC) of index cases with bacteriologically-confirmed pulmonary TB (HHC screening cohort), has been described^6^. The second, facility-based triage cohort (Fig. 1B) includes patients presenting at healthcare facilities with presumptive TB, all of whom were symptomatic (Clinic Triage Cohort).

For all participants, symptom screening, chest radiography (CXR), and spontaneously-expectorated sputum for Xpert MTB/RIF Ultra (Cepheid, CA, USA), liquid culture (Mycobacteria Growth Indicator Tube [MGIT], BACTEC, Beckton Dickinson, NJ, USA), and smear microscopy, were performed at baseline, regardless of symptom or CXR status. Blood was collected in PAXgene Blood RNA tubes (PreAnalytiX, Hombrechtikon, Switzerland) for RNA analysis and BD Vacutainer serum-separating tubes (Franklin Lakes, NJ, United States) for serum analysis. A participant was classified symptomatic if they reported one or more of cough, fever, weight loss, fatigue, night sweats, pleuritic chest pain, or haemoptysis, for any duration. CXRs were read once by an experienced investigator at each site using a standardized form and recorded as normal or abnormal (compatible with pulmonary TB), based on the absence or presence of any cavitation, opacity, mediastinal or hilar adenopathy, pleural effusion, bronchiectasis, or collapsed lung.

Bacteriologically-confirmed pulmonary TB disease was defined as one or more sputum specimens positive by MGIT culture or Xpert Ultra (excluding trace positive). Sputum induction was not performed. Participants who were unproductive of sputum, or with Xpert Ultra trace positive result only, were considered sputum-negative in the primary analysis. CXR results were not considered as part of the TB case definitions. Participants who, after investigation for TB at baseline, were not confirmed by positive sputum MGIT culture or Xpert Ultra, were defined as controls for the purpose of diagnostic analyses. Prevalent TB was defined as cases diagnosed on sputum samples collected at the baseline visit. Symptomatic and asymptomatic TB were defined as bacteriologically-confirmed prevalent TB with or without reported symptoms, respectively. All participants diagnosed with TB disease were referred for treatment.

### Community screening cohort (CORTIS)

The CORTIS study has been described in ref. ^4^. Briefly, we performed community-based, universal sputum screening of HIV-uninfected adults residing in TB-endemic communities in South Africa, as for RePORT SA (Fig. 1C). The study enrolled 2923 participants, who were pre-screened to enrich the study population for individuals who tested positive for the RISK11 transcriptomic signature. Prevalent TB diagnosed at baseline was microbiologically confirmed in at least two sputum samples.

### Case-control design

Biomarker analyses were performed on case-control subsets. For all cohorts, all TB cases were included (Fig. 1). For the HHC screening cohort, two controls were selected for each TB case by propensity score matching based on site, sex, age, and HIV status (Fig. 1A). For the clinic triage cohort, one and two controls were selected for each TB case for the RNA-seq and proteomic analyses, respectively, based on site, sex, age, BMI and HIV status (Fig. 1B). For the community screening cohort, three controls were selected for each prevalent TB case by propensity score matching based on site, sex and age (Fig. 1C and Supplementary Methods). The different ratios of cases to controls were informed primarily by study design, but operational feasibility and available funding at the time of the analyses was also considered.

### RNA-sequencing

For RePORT SA, participant RNA was extracted manually from PAXgene tubes using the PAXgene blood RNA kit (PreAnalytiX, Hombrechtikon, Switzerland). For CORTIS participants, RNA was extracted using a high-throughput, standardized, fully automated procedure on the Freedom EVO 150 robotic platform (Tecan, Männedorf, Switzerland) with the Maxwell SimplyRNA kit (Promega, Madison, WI, USA). RNA was treated using the NEBNext Globin & rRNA Depletion Kit (New England Biolabs, Ipswich, MA, United States) and sequenced on the DNBEQ-G400 sequencing platform, yielding at least 30 million paired-end 100-base reads per sample.

### Adolescent cohort study: progressor and non-progressor cohorts

Publicly available whole blood RNA-seq data from M.tb-infected TB progressors and non-progressors who participated in the longitudinal Adolescent Cohort Study were retrieved (GEO accession GSE79362). As described previously^29,42^, 6363 adolescents aged 12–18 years from the Western Cape, South Africa, were enrolled at high schools and followed for 24 months or more. Blood, including whole blood in PAXgene tubes, was collected at 6-monthly intervals. Evidence of M.tb infection was based on a positive QuantiFERON TB GOLD In-Tube Assay (QFT) and/or tuberculin skin test (TST). A total of 40 progressors with initial M.tb infection developed microbiologically confirmed incident TB disease >6 months after study enrollment. TB investigation was triggered by respiratory symptoms, reported household contact or TST/IGRA conversion^46^. Amongst those with M.tb infection who remained TB-free, 104 non-progressors who matched progressors by age, gender, ethnicity, school and prior history of TB, were selected^29,42^. ACS progressor data were aligned to each individual’s time of TB diagnosis and modeled using a smoothing spline.

### Blood transcriptome analyses

#### RNA-seq data processing and differential expression analysis

Raw sequencing reads were quality-checked using FastQC^47^ and MultiQC^48^. Trim-RNA Galore was used to remove adapters and filter raw reads below 36 bases long, and leading and trailing bases below quality 20. The filtered reads were aligned to the *Homo sapiens* reference genome (GRCh38.p14) using the HISAT2 v2.2.1 aligner^49^. The aligned reads were quantified to obtain gene-level counts using featureCounts from Subread v2.1.0^50^. Lowly expressed genes with counts per million (CPM) >2 in at least five samples were retained. Differentially expressed genes (DEGs) were identified using DESeq2, applying a false discovery rate (FDR) threshold of <0.05 and an absolute log_2_ fold change >1. For subsequent analyses requiring normalized expression, voom transformation was applied to the raw count data to yield log_2_-normalized expression data (Supplementary Methods: *“*RNA-seq pre-processing and batch-adjustment*”*).

### Molecular distance to health

We calculated molecular distance to health (MDH) scores, which quantified the deviation from a sample’s gene expression profile from a healthy reference population, as previously described^51^. In summary, we filtered the normalized gene expression and retained the 75% most variable genes, and calculated the median and standard deviation of the gene expression for the healthy control samples. Next, a z-score normalization was performed using the control samples as a reference. Absolute values of the normalized z-scores were obtained, and values above a threshold of 2 was set to zero. Finally, MDH scores were calculated as the average of these absolute z-scores for the perturbed genes in a sample.

### Pathway enrichment

To identify differentially regulated pathways, we performed differential pathway analysis using quantitative set analysis of gene expression (QuSAGE^52^) with blood transcription modules (BTM) gene sets representing diverse biological contexts. To complement the pathway analysis, we also employed gene co-expression network analysis using CEMiTool^27^ to identify sets of coherently expressed gene modules and performed overrepresentation analysis (ORA) for genes within each module using the clusterProfiler R package^53^. Pathways with FDR-adjusted *p* values <0.05 were considered significantly enriched.

### Measurement of serum proteins

Serum proteins were measured using Bio-Plex multiplex bead arrays on the Bio-Plex platform (Bio-Rad Laboratories, CA, USA), as previously described^54^. Samples from the RePORT SA HHC Screening and Clinic Triage cohorts were assayed using the Bio-Plex Pro Human Cytokine 27-Plex Panel and the Bio-Plex Pro Human Apolipoprotein 10-plex Assay Panel. Samples from the CORTIS Community Screening cohort were assayed using the Bio-Plex Pro Human Cytokine, Chemokine, and Growth Factor 48-Plex Panel.

### Machine learning for biomarker discovery

To identify a novel host transcriptomic signature of asymptomatic TB, we applied an in-house machine learning pipeline—OmicScan (https://github.com/SATVILab/OmicScan; DOI: 10.5281/zenodo.20041963)—to analyse the combined datasets from the HHC Screening and Community Screening cohorts. We split the combined dataset into a signature discovery set (75%) and a hold-out validation set (25%), and then further partitioned the discovery set into training (20%) and test (20%) sets (see Supplementary Methods for details).

The OmicScan pipeline included gene prefiltering to remove noisy/uninformative genes, gene ranking to retain the top outcome-associated genes, and derivation of the parsimonious biomarker model. Next, we applied four machine learning (ML) algorithms—Support Vector Machine (SVM), Random Forest (RF), Gradient Boosting (XGBoost) and K-Nearest Neighbor (KNN)—to train predictive models and subsequently evaluated their performance on the discovery test set. We used a “g-score”, a weighted combination of SHAP (Shapley Additive exPlanations) value and permutation importance score, to rank genes from each ML model based on their contribution to the predictive model. Together, the g-score determined the weighted usefulness of the gene. We note that the combined g-score is intended for use as a robust ranking heuristic rather than a calibrated or causal attribution measure, as SHAP values and permutation importance quantify complementary but heterogeneous aspects of model contribution. Accordingly, the g-score was used solely for relative gene prioritization and not for effect size interpretation. We further note that correlated gene sets (e.g., coregulated immune pathways) may have influenced importance estimates across both metrics.

Finally, we performed a greedy forward search on the top 10 ranked genes to derive the most predictive, parsimonious combination of genes for diagnostic application (Supplementary Methods).

### Integration of transcriptomic and proteomic data to discover clusters

We sought to integrate the abundance of serum proteins and blood RNA-seq datasets from asymptomatic TB cases from the two Screening cohorts to understand the heterogeneity amongst this TB phenotype. We employed similarity network fusion (SNF) in SNFTool^55^ to integrate transcriptomic and proteomic data modalities, allowing unsupervised identification of molecular subgroups within the asymptomatic TB dataset. Optimal cluster numbers were identified (2 to 8 clusters) by eigen-gap and rotation cost methods^55^. After identifying subclusters, we utilized the *clValid* R package^56^ to assess cluster stability and validity through several metrics, including the average proportion of non-overlap (APN), the average distance between means (ADM), and the adjusted Rand Index (ARI) (Supplementary Methods).

To explore the clinical relevance of the subclusters identified, subcluster membership was tested for association with TB-related clinical features, including MGIT liquid culture time to positivity (TPP), Xpert Ultra classification, sputum smear microscopy grade, CXR classified as abnormal, Qure.ai CXR score (Bengaluru, Karnataka, India), body-mass index (BMI), and IFN-γ levels obtained for TBAg2 tubes in the QuantiFERON-TB Plus (QIAGEN, Hilden, Germany) IGRA. Group comparisons were made using the Wilcoxon rank-sum test for continuous variables and the Fisher exact test for categorical variables.

### Reporting summary

Further information on research design is available in the Nature Portfolio Reporting Summary linked to this article.

## Supplementary information

Supplementary materials

Reporting summary

Transparent Peer Review file

## Data Availability

Deidentified, processed gene expression data and raw fast data has been deposited on the GEO repository (accession PRJNA1364747). Luminex data is available on ZivaHub at https://figshare.com/s/627c2e24299911aa77ca. Other source data are provided in the supplementary sections.
