# Supplementary material for: Inflammatory biomarkers of asymptomatic and symptomatic tuberculosis
Source: Nat Commun. 2026 Jul 25;17:9079. doi: 10.1038/s41467-026-75909-6 (PMC13518882; doi:10.1038/s41467-026-75909-6)
Supplement: Supplementary file 1 [file 41467_2026_75909_MOESM1_ESM.pdf]

**SUPPLEMENTARY INFORMATION**

**Inflammatory Biomarkers of Asymptomatic and Symptomatic Tuberculosis**

Denis Awany, Dominique T Ariefdien, Simon C Mendelsohn, Virginie Rozot, Humphrey Mulenga, Sarah Nyangu, Michele Tameris, Tumelo Moloantoa, Austin Katona, Fernanda Maruri, Firdows Noor, Ravindre Panchia, Khuthadzo Hlongwane, Kim Stanley, Yuri F van der Heijden, Katie Hadley, Andrew-Fiore Gartland, Craig Innes, William Brumskine, Keertan Dheda, Shameem Jaumdally, Tahlia Perumal, Neil Martinson, Al Leslie, Bernard Fourie, Andriëtte Hiemstra, Stephanus T Malherbe, Gerhard Walzl, Kogieleum Naidoo, Gavin Churchyard, Novel N Chegou, Timothy R Sterling, and Mark Hatherill, Thomas J Scriba, on behalf of the RePORT South Africa Study and the CORTIS Study Teams

**METHODS** **2**

Propensity Score matching for Case-Control Selection 2

RNA-seq pre-processing and batch-adjustment 2

Cohort-specific WGCNA to assess robustness to cohort integration and batch correction 3

Comparison of XpertHR transcriptomic signature across the subclusters of asymptomatic TB and validation in ACS data 3

Comparison of MDH scores across subclusters and validation in ACS data 4

Machine Learning for Biomarker Discovery 4

Integration of transcriptomic and proteomic data to discover clusters 5

SUPPLEMENTARY TABLES 7

SUPPLEMENTARY FIGUES 9

References 24

## Methods

### *Propensity Score matching for Case-Control Selection*

Propensity score matching was used to select controls that are comparable to cases with respect to prespecified covariates. Propensity scores were estimated using logistic regression, in which case-control status was modelled as a function of sex, age, and HIV status, and study site. No additional covariates were included in the propensity score model.

Matching was performed using nearest-neighbour matching based on the estimated propensity scores. For each of the HHC Screening and Community Screening cohorts, each case was matched to 3 controls with the closest propensity score, whereas each case was matched to 2 controls for the Clinic Triage cohort. Selection was conducted without replacement and without imposing a calliper restriction. This approach was chosen to maximize retention of eligible cases while improving comparability between cases and controls with respect to study site, sex, age, and HIV status. Following matching, covariate balance between cases and controls was evaluated using standardized mean differences and empirical cumulative distribution function comparisons.

### *RNA-seq pre-processing and batch-adjustment*

Gene-level count matrices from the Clinic Triage cohorts (RePORT-SA 2A), HHC Screening (RePORT-SA 2B), and Community Screening (CORTIS) were merged based on shared gene identifiers. Prior to batch correction, lowly expressed genes were filtered to reduce technical noise and improve statistical power, by retaining genes if they exhibited counts per million (CPM) greater than 1 in at least 10 samples across the combined dataset.

Batch effects arising from differences in cohort origin were corrected using the ComBat-seq algorithm[1] implemented in the Bioconductor R package. Following batch correction, gene expression data were normalized to account for differences in library size and sequencing depth. The normalized count data were subsequently transformed using the voom method[2] implemented in the limma R package, and generated log2-transformed expression values.

The same pre-processing and batch correction procedure was performed when merging and analysing the combined dataset from the HHC Screening (RePORT-SA 2B), Community Screening (CORTIS), and the Adolescent Cohort Study progressor and non-progressor sub-study (ACS, see below).

The effectiveness of batch correction was evaluated using principal component analysis (PCA) before and after adjustment. Prior to batch correction, PCA revealed clear separation of samples according to cohort, indicating the presence of batch-associated technical variation (**Supplementary**

**Figures 11** and **12**). Following ComBat-seq correction, samples no longer clustered by cohort and instead showed improved integration across datasets, with no discernible batch-specific structure in the data distribution (**Supplementary Figures 11** and **12**). These results demonstrate that the batch correction procedure effectively mitigated cohort-driven technical effects.

### *Cohort-specific WGCNA to assess robustness to cohort integration and batch correction*

To evaluate whether the WGCNA module structure and module-phenotype associations identified in the integrated dataset were influenced by batch correction or cohort integration, we repeated the WGCNA analysis independently for each cohort (HHC Screening, Community Screening, and Clinic Triage cohort). For each cohort, normalized gene expression data were analyzed separately without combining samples across cohorts. Gene co-expression network analysis using CEMiTool[3], using the top 75% most variable genes, identified 6, 4, and 7 modules in the HHC Screening, Community Screening, and Clinic Triage cohort, respectively. Module enrichment patterns were evaluated across the TB disease groups within each cohort.

The observed module-disease associations and their biological pathway annotation are consistent with those observed in the integrated analysis. In particular, modules corresponding to interferon signalling (M3 in the combined cohort analysis), and protein synthesis (M4 in the integrated analysis) exhibited progressive increases in enrichment scores across the TB disease spectrum, from no-TB controls to asymptomatic TB and symptomatic TB. Similarly, lymphocyte modules such as B cell activation and signalling and T cell signalling were decreased in the HHC Screening cohort, Community Screening cohort, and the Clinic Triage cohort. The complement activation module did not show a consistent increase or decrease in the three individual cohorts (**Supplementary Figure 13**).

### *Comparison of XpertHR transcriptomic signature across the subclusters of asymptomatic TB and validation in ACS data*

To contextualize the transcriptional changes observed in asymptomatic TB, we compared transcriptomic signature scores derived from the community cohorts (cross-sectional data from the HHC Screening [RePORT SA] and Community Screening [CORTIS] cohorts) with longitudinal transcriptomic data from the Adolescent Cohort Study (ACS), a prospective cohort in which individuals were followed over time. A subset of individuals with evidence of Mtb infection was subsequently diagnosed with TB (progressors), while another subset remained TB-free despite Mtb infection (non-progressors)[4, 5]. Prior to analysis, gene expression data from three cohorts were merged based on matching by commonly expressed genes and pre-processed as described above (see *RNA-seq pre-processing and batch-adjustment*). In the ACS progressors, sample collection timepoints were realigned relative to the date of TB diagnosis, such that time was expressed as days prior to diagnosis, as described[4, 5].

A smoothing spline model was fitted to transcriptomic signature scores in ACS progressors as a function of time to diagnosis to estimate the continuous trajectory of transcriptional perturbation during progression to TB. The median signature score among asymptomatic TB cases in the combined community cohorts was then overlaid onto this trajectory. The point of intersection between the community cohorts median score and the ACS spline curve was used to estimate the approximate pre-diagnostic time at which the molecular state of asymptomatic TB in the community cohort corresponded to that observed during TB progression in the ACS.

### *Comparison of MDH scores across subclusters and validation in ACS data*

To further validate the robustness of the XpertHR signature results using an independent approach, molecular distance to health (MDH) scores were computed on the combined and batch-corrected gene expression dataset comprising the HHC Screening, Community Screening, and ACS progressor and non-progressor sub-study cohorts. For this calculation, all TB progressors from ACS were treated as cases and non-progressors as the control reference population.

To ensure that the observed MDH patterns were not influenced by imbalance in the number of control samples within and across cohorts, we proceeded as follows: we identified the cohort with the smallest number of controls (which was the ACS with 99 controls) and randomly subsampled an equal number of controls from the larger cohorts, including the HHC Screening, and Community Screening. For the two cohorts with larger control pools, bootstrap resampling was performed over 100 iterations, each time randomly selecting the same number of controls as in the smallest cohort and recalculating MDH scores on the merged dataset (with cases from all three cohorts, all controls from ACS, and selected controls from HHC Screening and Community Screening cohorts) but only using the selected controls. Median MDH values across bootstrap iterations were used for downstream comparisons.

The median MDH score observed among asymptomatic TB cases in the community cohort was then compared to the spline-modelled MDH trajectory from the ACS, with MDH scores for ACS being plotted against time to TB diagnosis for progressors and a smoothing spline model fitted to characterize the temporal trajectory of global transcriptional perturbation during progression. The intersection point between the community cohort median MDH value and the ACS spline curve was used to estimate the corresponding time prior to TB diagnosis at which similar molecular perturbation occurs during TB progression.

### *Machine Learning for Biomarker Discovery*

To identify a novel host transcriptomic signature of asymptomatic TB, we applied an in-house machine learning pipeline—OmicScan (https://github.com/SATVILab/OmicScan)—to analyse the combined dataset of 397 samples, comprising 46 asymptomatic TB and 144 controls from the HHC Screening and 27 asymptomatic TB and 180 controls from the Community Screening cohorts. We

split the combined dataset into a signature discovery set (75%) and a hold-out validation set (25%), and then further partitioned the discovery set into training (20%) and test (20%) sets. We employed stratified sampling to ensure that the splits obtained were not imbalanced with respect to cohort representation and the proportion of the class labels. Each set was independently batch-corrected using ComBat-Seq[1].

The OmicScan pipeline included gene prefiltering to remove noisy/uninformative genes, gene ranking to retain the top outcome-associated genes, and derivation of the parsimonious biomarker model. For gene prefiltering, we applied (1) the t-test with Benjamini-Hochberg procedure to control the false discovery rate (FDR) and retained genes with adjusted p-value (FDR) <0.05, and (2) Hedge's g as the measure of effect size (ES) and retained genes with an ES greater than 0.3. Next, we applied four machine learning (ML) algorithms—Support Vector Machine (SVM), Random Forest (RF), Gradient Boosting (XGBoost) and K-Nearest Neighbour (KNN)—to train predictive models and subsequently evaluated their performance on the discovery test set. We used a "g-score", a weighted combination of SHAP (Shapley Additive exPlanations) value and permutation importance score, to rank genes from each ML model based on their contribution to the predictive model. Permutation importance provides a model-agnostic estimate of feature relevance by measuring the decrease in model performance when a feature's value is randomly shuffled. This captured the global impact of each gene on model accuracy[6]. In contrast, SHAP values offered a local interpretability framework quantifying the contribution of each gene to individual predictions based on cooperative game theory[7]. Together, the g-score determined a weighted usefulness of the gene.

Finally, we performed a greedy forward search on the top 10 ranked genes to derive the most predictive, parsimonious combination of genes for diagnostic application. We assessed the diagnostic power of every possible combination of the genes by calculating signature scores of each combination and the corresponding area under the receiver operating characteristic curve (AUROC). For gene combinations containing both up- and down-regulated genes, the signature score was calculated by subtracting the mean expression of the down-regulated genes from that of the up-regulated genes. In contrast, for combinations in which all genes were regulated in the same direction, the signature score was derived using the geometric mean of the expression levels of the associated genes.

***Integration of transcriptomic and proteomic data to discover clusters***

We sought to integrate the abundance of serum proteins and blood RNA-seq datasets from asymptomatic TB cases from the two community screening cohorts to understand the heterogeneity amongst this TB phenotype. We employed Similarity Network Fusion (SNF) in SNFTool[8] to integrate transcriptomic and proteomic data modalities, allowing unsupervised identification of molecular

subgroups within the asymptomatic TB dataset. Optimal cluster numbers were identified (2 to 8 clusters) by eigen-gap and rotation cost methods[8].

Given that SNF performance can be sensitive to tuning parameters - particularly the number of nearest neighbours (K) used to construct sample similarity graphs and the similarity kernel scaling parameter (alpha) - we implemented a systematic parameter optimization framework rather than relying on default settings. We performed grid-search-based tuning across a range of K values (from 10 to 50, which contains the square root of the sample size) and alpha values (alpha ranging from 0.2 to 0.5, consistent with published recommendations), with the number of fusion iterations fixed to 50. For each candidate parameter combination, SNF was trained within a cross-validation framework. Specifically, the dataset was partitioned into cross-validation folds, SNF networks were constructed on the training folds, and clustering was performed using spectral clustering. Cluster quality was then evaluated on held-out samples using the average silhouette width as an objective metric of cluster separation and cohesion. This procedure was repeated iteratively across parameter combinations and resampling iterations (100 repetitions), and the optimal K and alpha values were selected as the configuration with the highest average cross-validated silhouette score.

Given the high heterogeneity and dimensionality of the multiomic data, evaluating cluster validity was essential to minimize the risk of identifying spurious clusters that may arise from algorithmic bias. After identifying subclusters, we utilized the *clValid* R package[9] to assess cluster stability and validity through several metrics, including the average proportion of non-overlap (APN), the average distance between means (ADM), and the adjusted Rand Index (ARI). The APN quantified the extent to which observations changed cluster membership when a subset of features is systematically removed. Specifically, it calculated the average proportion of observations that were assigned to different clusters in a perturbed dataset compared to the original clustering. APN values ranged from 0 to 1, with lower values indicating greater cluster stability under feature perturbation. ADM evaluated the average Euclidean distance between cluster centroids obtained from the original dataset and those obtained after feature removal. ADM values ranged from 0 to 1, with smaller values suggesting that the overall cluster remained consistent across perturbations. ARI, a statistic ranging from 0 (no agreement) to 1 (perfect concordance) measured similarity between two clustering assignments (original and perturbed). Cluster stability was evaluated by performing 500 resampling iterations, each involving a random selection of 80% of the participant samples, in line with established methodological practices[10] (**Supplementary Figure 8**). To further examine the relationship between alternative sub-clustering results, we conducted chi-square tests to assess their statistical independence.

## Supplementary Tables

**Supplementary Table 1**. Distributions of age, sex, body mass index (BMI), and HIV status in participants from the Community Screening Cohort (top), HHC Screening Cohort (middle) and Clinic Triage Cohort (bottom).

**Community Screening Cohort (CORTIS)**

| | **No TB (N=180)** | **Asymptomatic TB (N=27)** | **Symptomatic TB (N=6)** | **Total (N=231)** |
|---|---|---|---|---|
| Median age (IQR) | 28 (23.00, 36.25) | 32 (25.00, 36.50) | 26 (24.25, 27.75) | 28 (23.00, 36.00) |
| Sex, n (%) | | | | |
| Female | 71 (39.4%) | 11 (40.7%) | 4 (66.7%) | 93 (40.3%) |
| Male | 109 (60.6%) | 16 (59.3%) | 2 (33.3%) | 138 (59.7%) |
| Median BMI (IQR) | 21.61 (19.42, 26.60) | 20.63 (18.48, 22.80) | 20.46 (19.44, 24.35) | 21.15 (19.19, 25.72) |
| HIV status, n (%) | | | | |
| Negative | 180 (100.0%) | 27 (100.0%) | 6 (100.0%) | 231 (100.0%) |

**HHC Screening Cohort (Report-SA)**

| | **No TB (N=227)** | **Asymptomatic TB (N=46)** | **Symptomatic TB (N=7)** | **Total (N=290)** |
|---|---|---|---|---|
| Median age (IQR) | 38.69 (26.07, 53.67) | 34.96 (25.79, 50.14) | 46.95 (33.44, 52.78) | 37.82 (26.28, 53.24) |
| Sex, n (%) | | | | |
| Female | 113 (49.8%) | 20 (43.5%) | 4 (57.1%) | 145 (50.0%) |
| Male | 101 (44.5%) | 17 (37.0%) | 3 (42.9%) | 122 (42.1%) |
| Unknown | 13 (5.7%) | 9 (19.6%) | 0 (0.0%) | 23 (7.9%) |
| HIV status, n (%) | | | | |
| Negative | 179 (78.9%) | 31 (67.4%) | 5 (71.4%) | 218 (75.2%) |
| Positive | 35 (15.4%) | 6 (13.0%) | 2 (28.6%) | 49 (16.9%) |
| Unknown | 13 (5.7%) | 9 (19.6%) | 0 (0.0%) | 23 (7.9%) |

**Clinic Triage Cohort (Report-SA)**

| | **No TB (N=212)** | **Asymptomatic TB (N=0)** | **Symptomatic TB (N=190)** | **Total (N=402)** |
|---|---|---|---|---|
| Median age (IQR) | 35.75 (29.10, 44.40) | | 35.2 (28.10, 42.25) | 35.45 (28.72, 43.92) |
| Sex, n (%) | | | | |
| Female | 136 (64.2%) | | 124 (65.3%) | 260 (64.7%) |
| Male | 76 (35.8%) | | 66 (34.7%) | 142 (35.3%) |
| Median BMI (IQR) | 23.6 (20.30, 27.20) | | 19.65 (17.60, 23.20) | 21.6 (18.70, 25.65) |
| HIV status, n (%) | | | | |
| Negative | 121 (57.1%) | | 103 (54.2%) | 224 (55.7%) |
| Positive | 85 (40.1%) | | 68 (35.8%) | 153 (38.1%) |
| Unknown | 6 (2.8%) | | 19 (10.0%) | 25 (6.2%) |

**Supplementary Table 2**. Distributions of sex, BMI, HIV status, and screening cohort across subclusters derived from asymptomatic TB (aTB (cluster 1), aTB (cluster 2)), as shown in **Figure 6,** as well as across participants without TB and those with symptomatic TB (sTB).

| | aTB (cluster 1) (N=26) | aTB (cluster 2) (N=29) | No TB (N=324) | sTB (N=13) | Total (N=392) | p value |
|---|---|---|---|---|---|---|
| HIV status | | | | | | 0.865 |
| − Negative | 24 (92.3%) | 26 (89.7%) | 295 (91.0%) | 11 (84.6%) | 356 (90.8%) | |
| − Positive | 2 (7.7%) | 3 (10.3%) | 29 (9.0%) | 2 (15.4%) | 36 (9.2%) | |
| BMI | | | | | | 0.108 |
| − Median | 21.8 | 19.4 | 21.6 | 20.5 | 21.1 | |
| − Q1, Q3 | 20.1, 25.6 | 18.1, 21.2 | 19.4, 26.6 | 19.4, 24.3 | 19.3, 26.0 | |
| Sex | | | | | | 0.376 |
| − N−Miss | 0 | 0 | 13 | 0 | 13 | |
| − Female | 14 (53.8%) | 11 (37.9%) | 135 (43.4%) | 8 (61.5%) | 168 (44.3%) | |
| − Male | 12 (46.2%) | 18 (62.1%) | 176 (56.6%) | 5 (38.5%) | 211 (55.7%) | |
| Cohort | | | | | | 0.723 |
| − Community Screening | 12 (46.2%) | 15 (51.7%) | 180 (55.6%) | 6 (46.2%) | 213 (54.3%) | |
| − HHC Screening | 14 (53.8%) | 14 (48.3%) | 144 (44.4%) | 7 (53.8%) | 179 (45.7%) | |

## Supplementary Figues

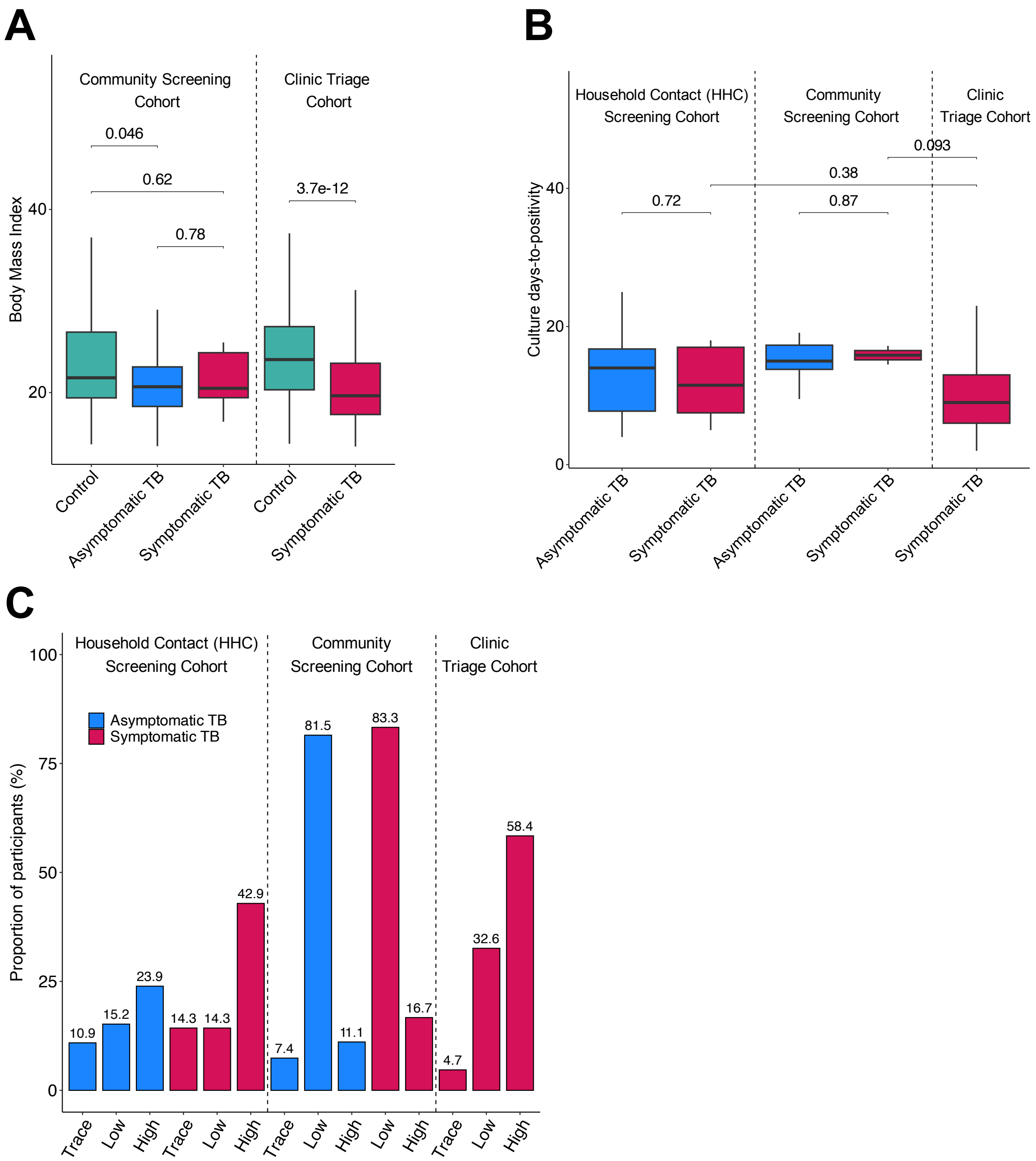


**Supplementary Figure 1. Distribution of clinical and microbiological characteristics of participants in the cohorts.** (**A**) Boxplot showing the distribution of body mass index (BMI) by clinical outcome in the Clinic Triage (no TB, n=209; symptomatic TB, n=182) and the Community Screening cohorts. (no TB, n=180; asymptomatic TB, n=27; symptomatic TB, n=6). $p$-values were computed using Mann-Whitney U test. For box-and-whisker plots horizontal lines indicate the median, the boxes the interquartile range (IQR) and the whiskers the range of data with $1.5 \times IQR$ from the lower and upper quartiles. (**B**) Boxplot showing time to sputum culture positivity (in days) for culture-diagnosed prevalent TB cases in the Clinic Triage (symptomatic TB, n=189), HHC Screening (asymptomatic TB, n=34; symptomatic TB, n=6), and the Community Screening cohorts (asymptomatic TB, n=21; symptomatic TB, n=2). $p$-values were computed with the Mann-Whitney U

test. For box-and-whisker plots horizontal lines indicate the median, the boxes the interquartile range (IQR) and the whiskers the range of data with $1.5 \times IQR$ from the lower and upper quartiles. (**C**) Bar chart showing the relative proportions of individuals in each Xpert semiquantitative category among asymptomatic TB (Community Screening, n=27; HHC Screening, n=46), and symptomatic TB (Community Screening, n=6; HHC Screening, n=7, Clinic Triage, n=182) cases in the cohorts. Proportions were calculated relative to the total number of individuals with each TB outcome, for those with available results. The category "Low" includes 'low' and 'very low', and "High" includes "medium" and "high" Xpert MTB/RIF Ultra categories.

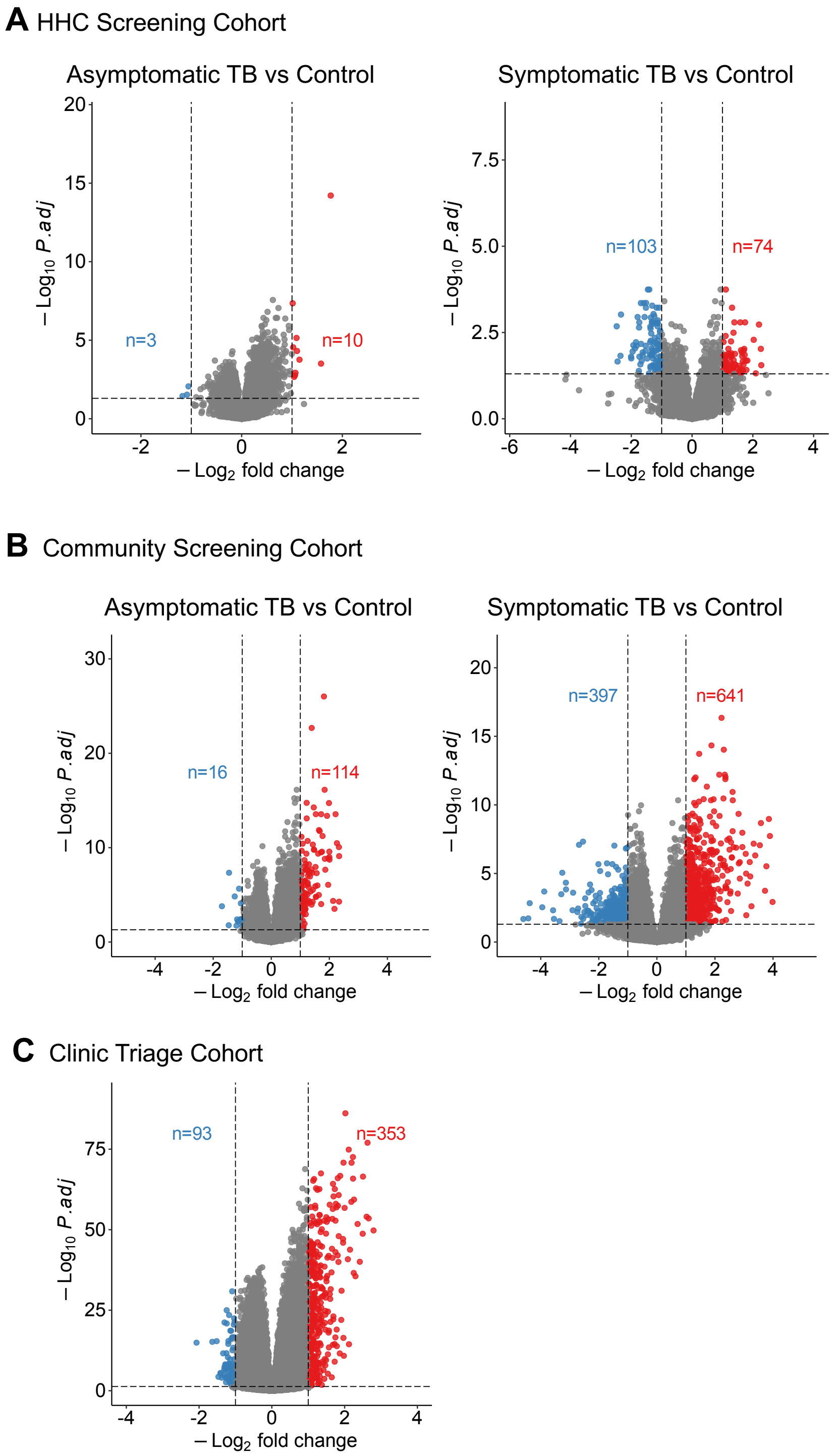


**Supplementary Figure 2. Analysis of differentially expressed genes between asymptomatic TB or symptomatic TB and TB-negative controls.** Volcano plots show the distributions of differentially expressed genes between asymptomatic TB and controls (no TB), or between symptomatic TB and controls for the HHC Screening (**A:** no TB, n=144; asymptomatic TB, n=46; symptomatic TB, n=7), Community Screening (**B**: no TB n=180, asymptomatic TB, n=27; symptomatic TB, n=6), and Clinic Triage (**C**: no TB, n=212; symptomatic TB, n=197) cohorts. The X-axis represents $log_2$ fold change, and the Y-axis represents $-log_{10}$ of the FDR-adjusted $p$-value. Genes with significant upregulation ($log_2$ fold change ≥ 1.0, FDR p < 0.05) are highlighted in red,

significant downregulation in blue, and non-significant genes in grey. Analysis performed using DESeq2 with Wald test.

A

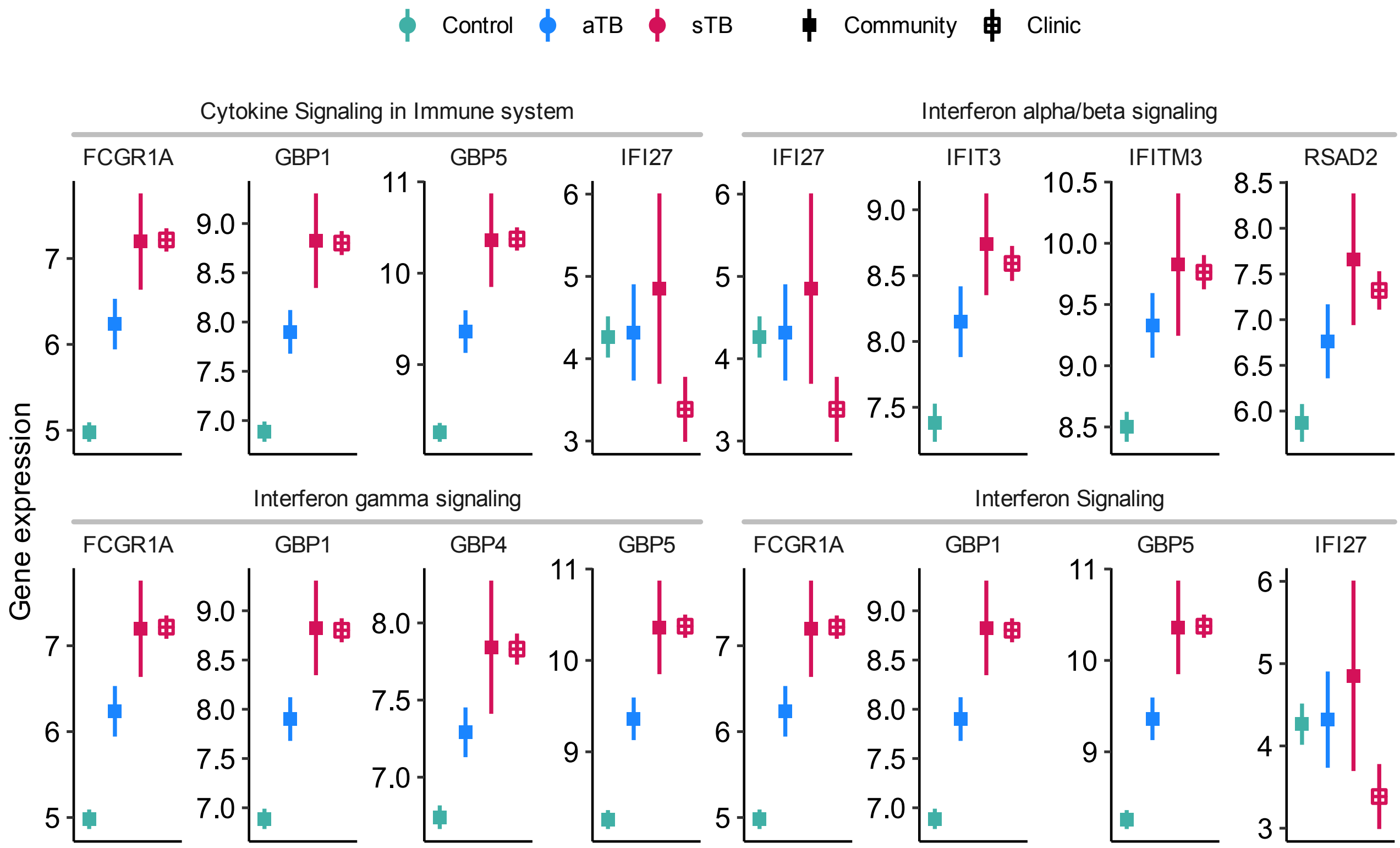


B

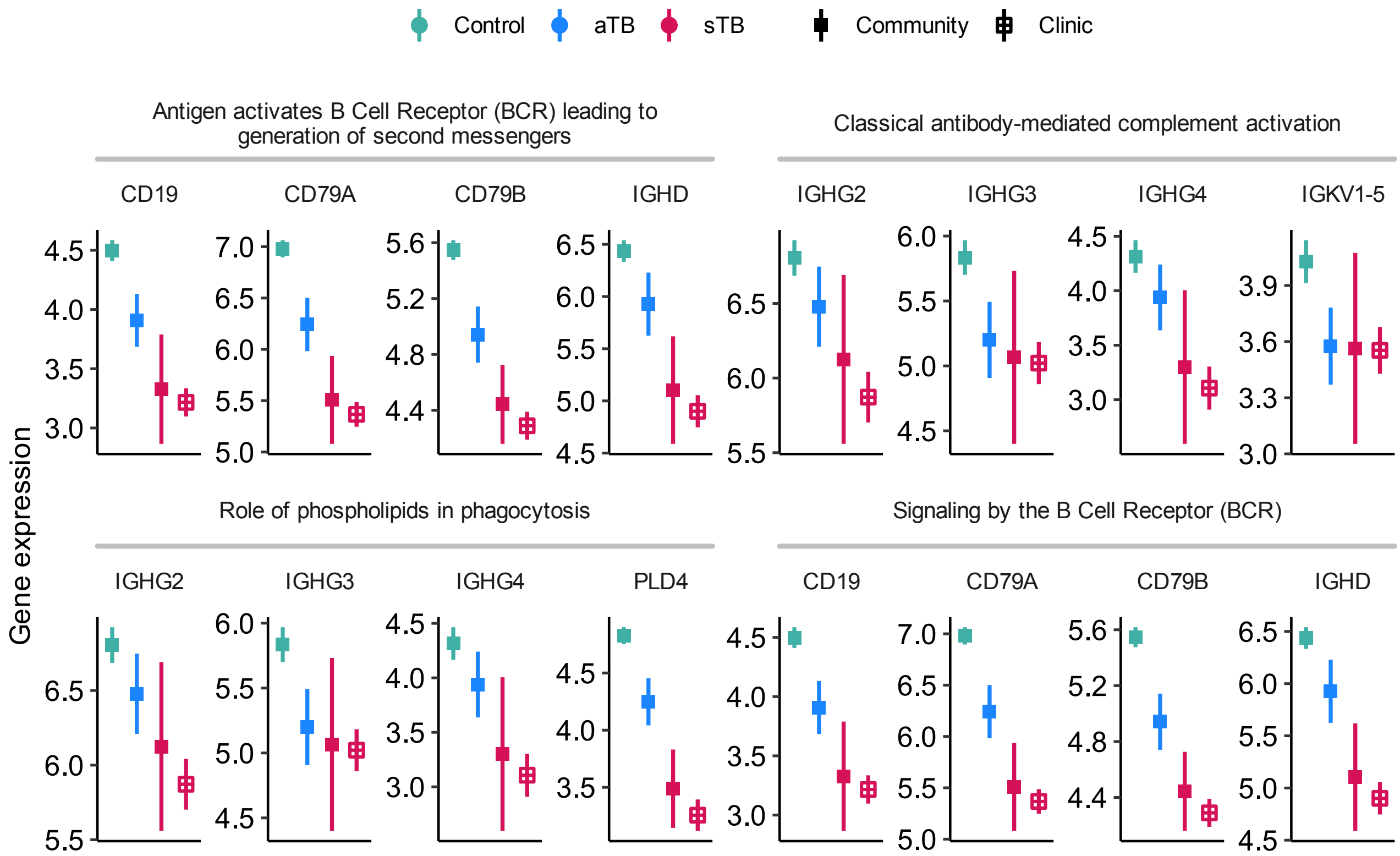

**Supplementary Figure 3. Analysis of relative expression levels for genes identified by gene co-expression network analysis across the TB spectrum.** Participants from the (no TB, n=144, asymptomatic TB, n=46; symptomatic TB, n=7) and Community Screening (no TB n=180, asymptomatic TB, n=27; symptomatic TB, n=6) and Clinic Triage cohorts (no TB, n=212; symptomatic TB, n=197) were stratified into groups along the TB spectrum, from healthy controls, asymptomatic TB (aTB) and symptomatic TB (sTB, further split into Screening and Clinic Triage), by weighted gene co-expression network analysis using CEMiTool. For these analyses, data from the HHC Screening (RePORT SA) and Community Screening (CORTIS) cohorts were combined. Gene set overrepresentation analysis was performed for genes in the 6 modules identified by weighted gene co-expression network analysis. Shown are the expression levels for the top four most regulated genes from each of the top 5 significantly enriched pathways in module M3 (**A**), module M5 (**B**).

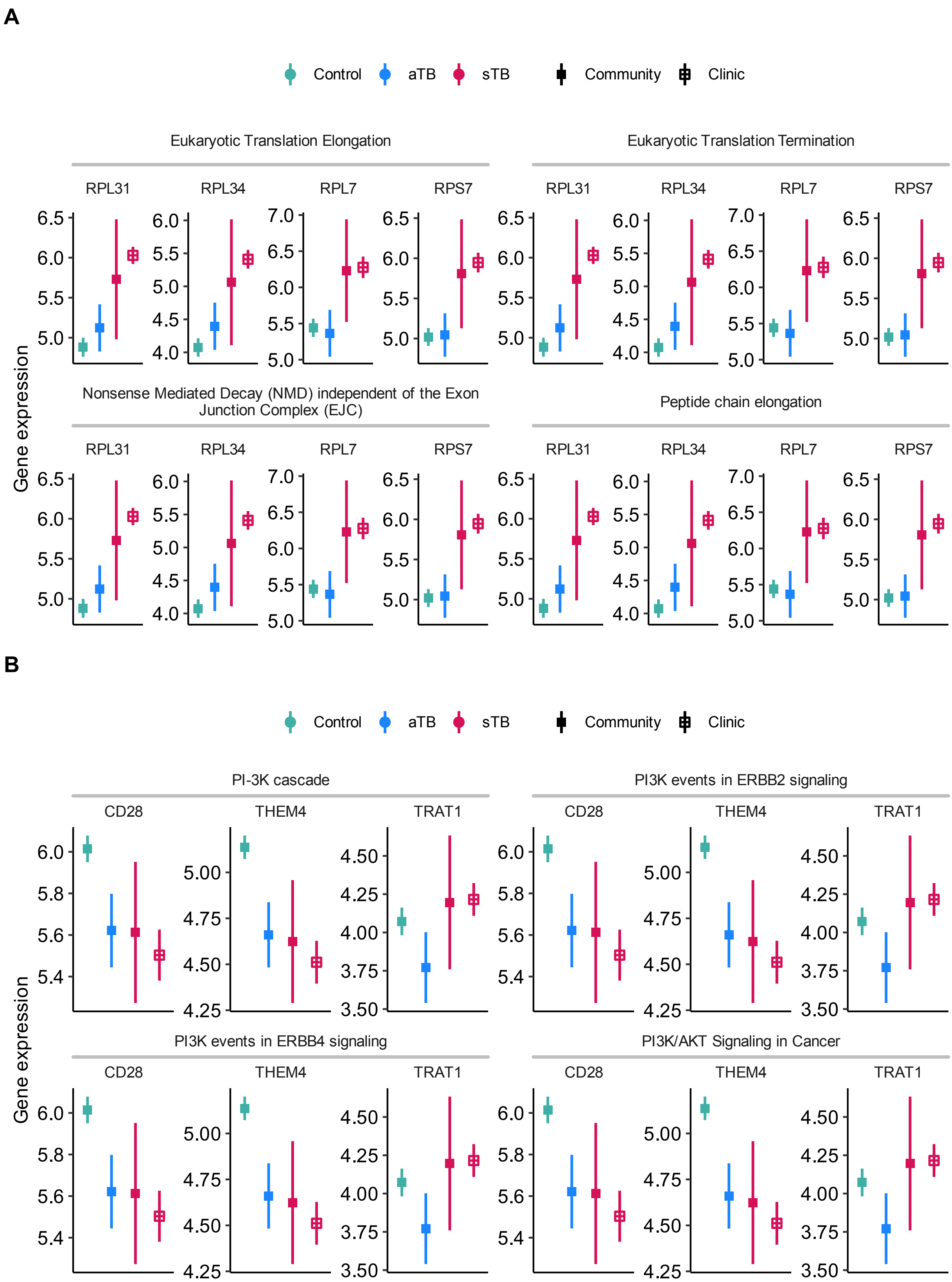


**Supplementary Figure 4. Analysis of relative expression levels for genes identified by gene co-expression network analysis across the TB spectrum.** Participants from the (no TB, n=144, asymptomatic TB, n=46; symptomatic TB, n=7) and Community Screening (no TB n=180, asymptomatic TB, n=27; symptomatic TB, n=6) and Clinic Triage cohorts (no TB, n=212;

symptomatic TB, n=197) were stratified into groups along the TB spectrum, from healthy controls, asymptomatic TB (aTB) and symptomatic TB (sTB, further split into Screening and Clinic Triage), by weighted gene co-expression network analysis using CEMiTool. For these analyses, data from the HHC Screening (RePORT SA) and Community Screening (CORTIS) cohorts were combined. Gene set overrepresentation analysis was performed for genes in the 6 modules identified by weighted gene co-expression network analysis. Shown are the expression levels for the top four most regulated genes from each of the top 5 significantly enriched pathways in module M4 (**A**), module M6 (**B**).

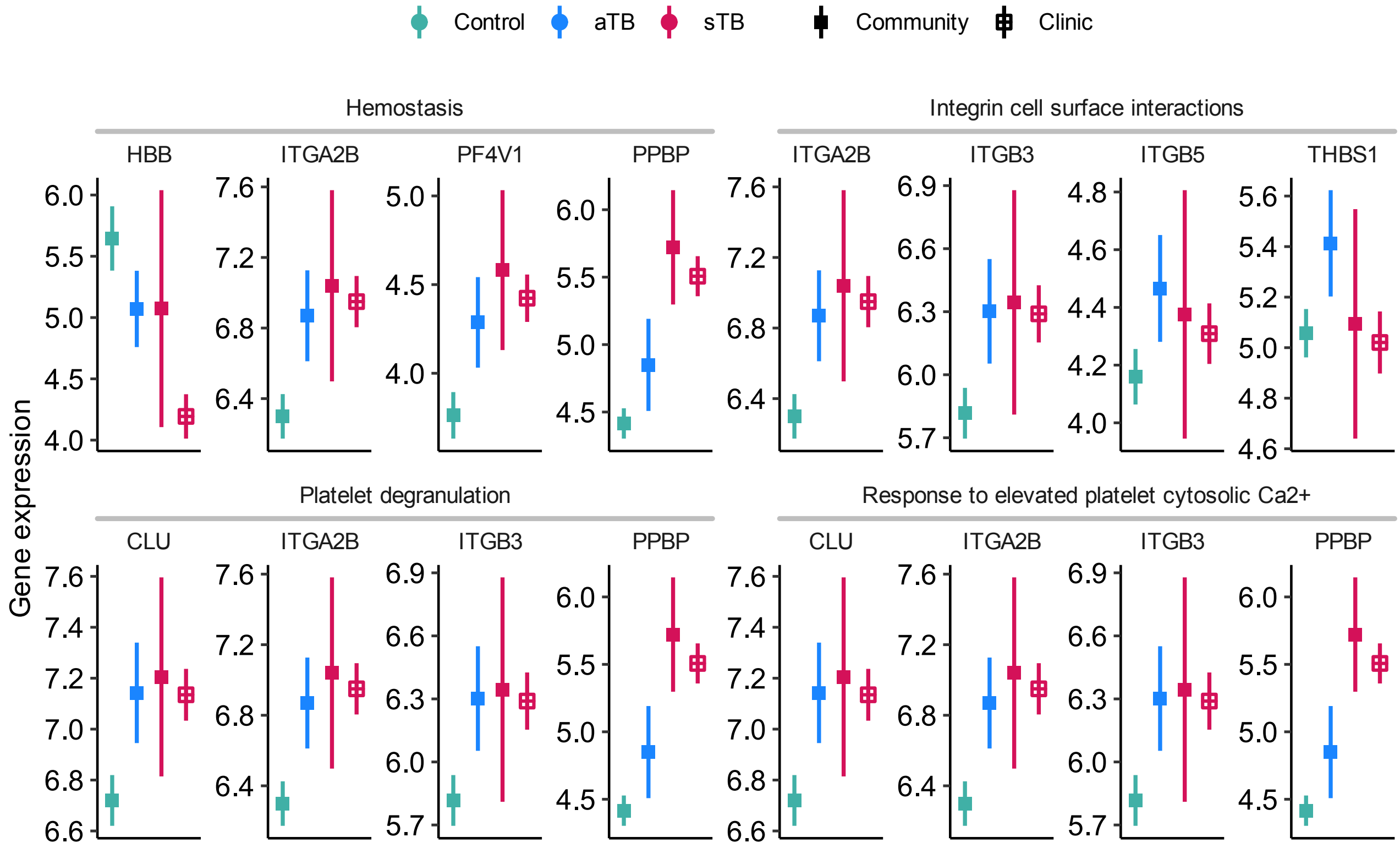


**Supplementary Figure 5. Analysis of relative expression levels for genes identified by gene co-expression network analysis across the TB spectrum.** Participants from the (no TB, n=144, asymptomatic TB, n=46; symptomatic TB, n=7) and Community Screening (no TB n=180, asymptomatic TB, n=27; symptomatic TB, n=6) and Clinic Triage cohorts (no TB, n=212; symptomatic TB, n=197) were stratified into groups along the TB spectrum, from healthy controls, asymptomatic TB (aTB) and symptomatic TB (sTB, further split into Screening and Clinic Triage), by weighted gene co-expression network analysis using CEMiTool. For these analyses, data from the HHC Screening (RePORT SA) and Community Screening (CORTIS) cohorts were combined. Gene set overrepresentation analysis was performed for genes in the 6 modules identified by weighted gene co-expression network analysis. Shown are the expression levels for the top four most regulated genes from each of the top 5 significantly enriched pathways in module M1.

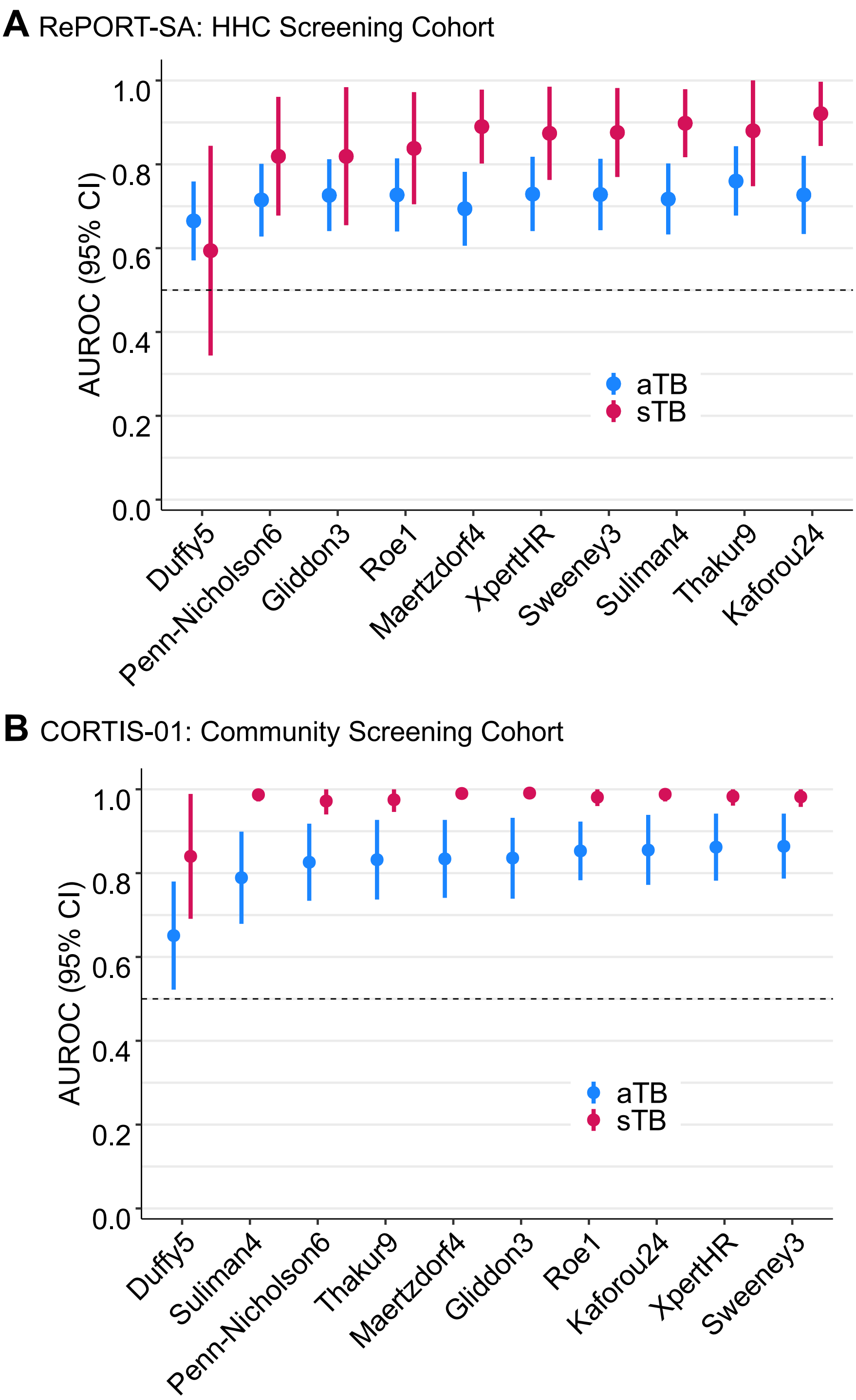


**Supplementary Figure 6. Evaluating diagnostic performance of existing, published transcriptomic biomarkers.** Area under the receiver-operating characteristic curve (AUROC) showing the performance of selected published transcriptomic signatures for differentiating between asymptomatic TB (aTB) and TB-negative controls, and between symptomatic TB (sTB) and TB-negative controls in the HHC Screening Cohort (**A**: no TB, n=144, asymptomatic TB, n=46; symptomatic TB, n=7), and in the Community Screening Cohort (**B**: no TB, n=180, asymptomatic TB, n=27; symptomatic TB, n=6). Shown are AUROC values (dots) and 95% CIs (error bars) for each signature.

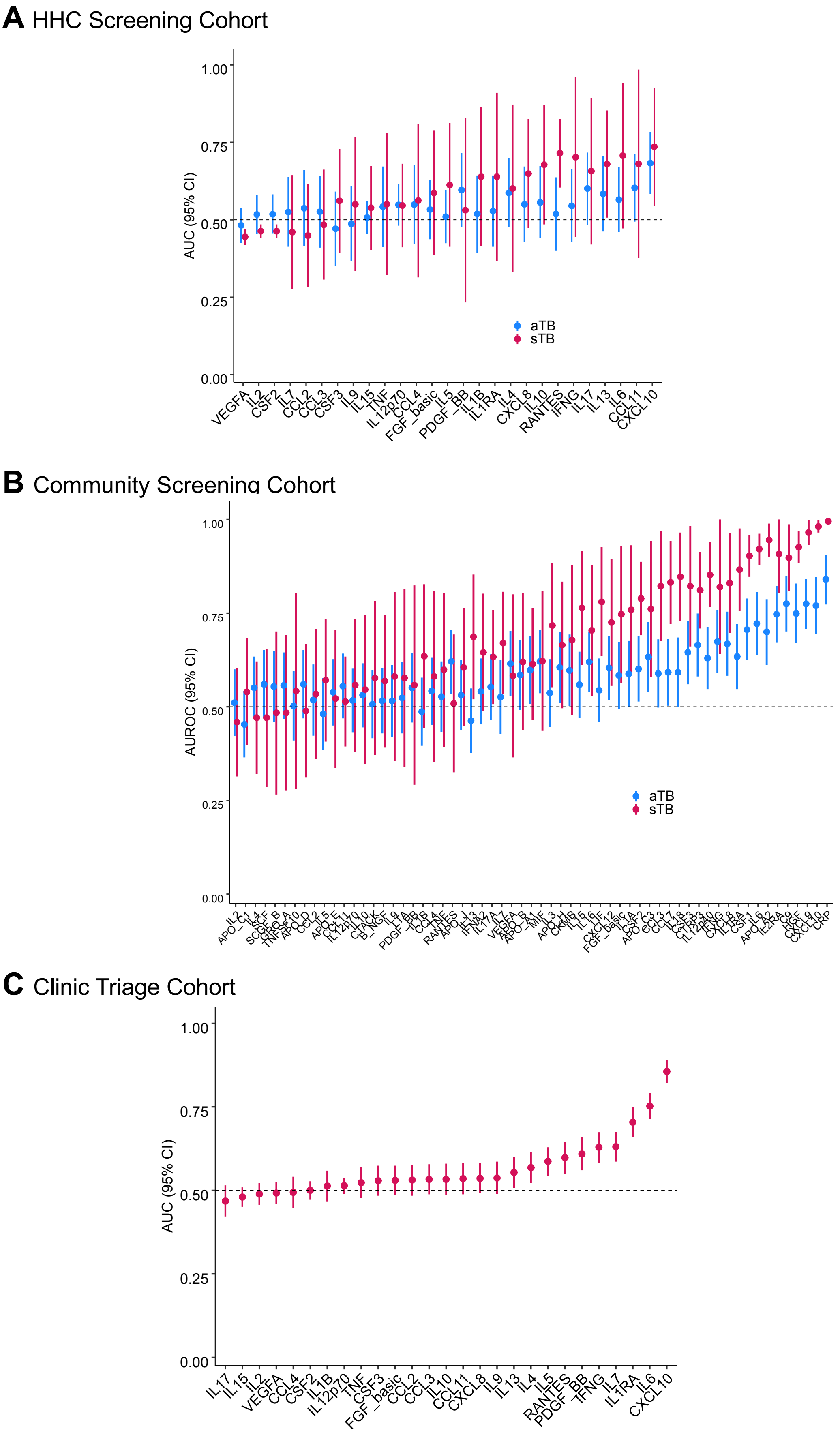


**Supplementary Figure 7. Evaluating diagnostic performance of serum proteomic markers.** Area under the receiver-operating characteristic curve (AUROC) showing the performance of serum

proteins measured by multiplex bead array in the cohorts. Diagnostic performance for differentiating between asymptomatic TB and TB-negative controls (blue), and between symptomatic TB and TB-negative controls (red) in the HHC Screening Cohort (**A**: no TB, n=136; asymptomatic TB, n=28; symptomatic TB, n=7), between asymptomatic TB and TB-negative controls, and between symptomatic TB and TB-negative controls in the Community Screening Cohort (**B**: no TB, n=167; asymptomatic TB, n=52; symptomatic TB, n=9), and between symptomatic TB and symptomatic TB-negative controls in the Clinic Triage cohort (**C**: no TB, n=460; symptomatic TB, n=206). Dots and error bars indicate the AUROC and 95% CIs.

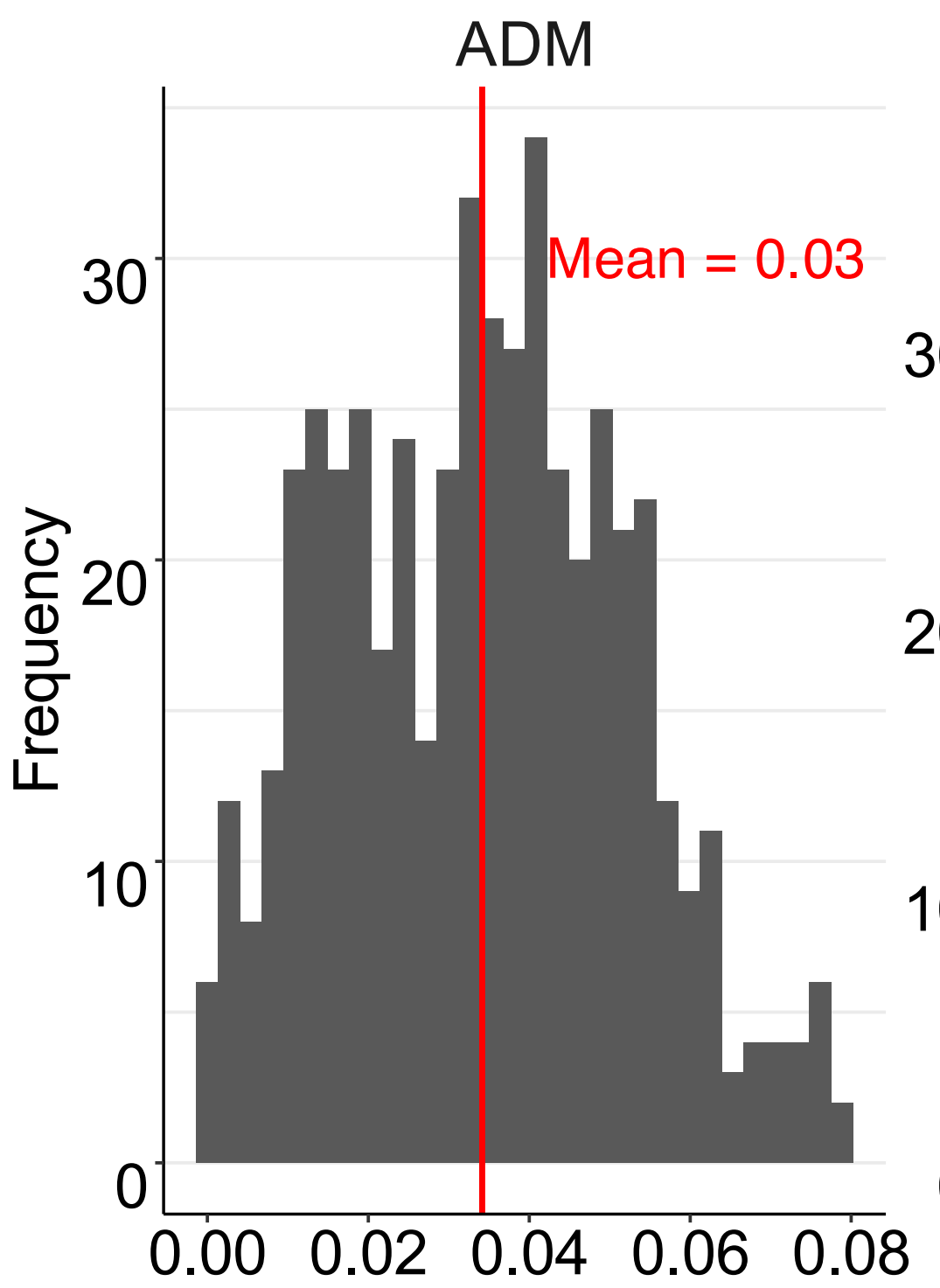


**Supplementary Figure 8. Stability analysis of asymptomatic TB clusters identified by fused similarity network analysis.** Two metrics were used to evaluate robustness of the two identified clusters among asymptomatic TB participants, estimated from 500 resampling iterations, each involving a random selection of 80% of the participant samples. (Left) Average Distance between Means (ADM), with a mean value of 0.03, indicates a small separation between resampled cluster centroids. (Right) Average Proportion of Non-overlap (APN), with a mean value of 0.07, reflects high cluster stability across resampling. Together, these low ADM and APN values suggest that the two subclusters identified by similarity network fusion are stable and reproducible.

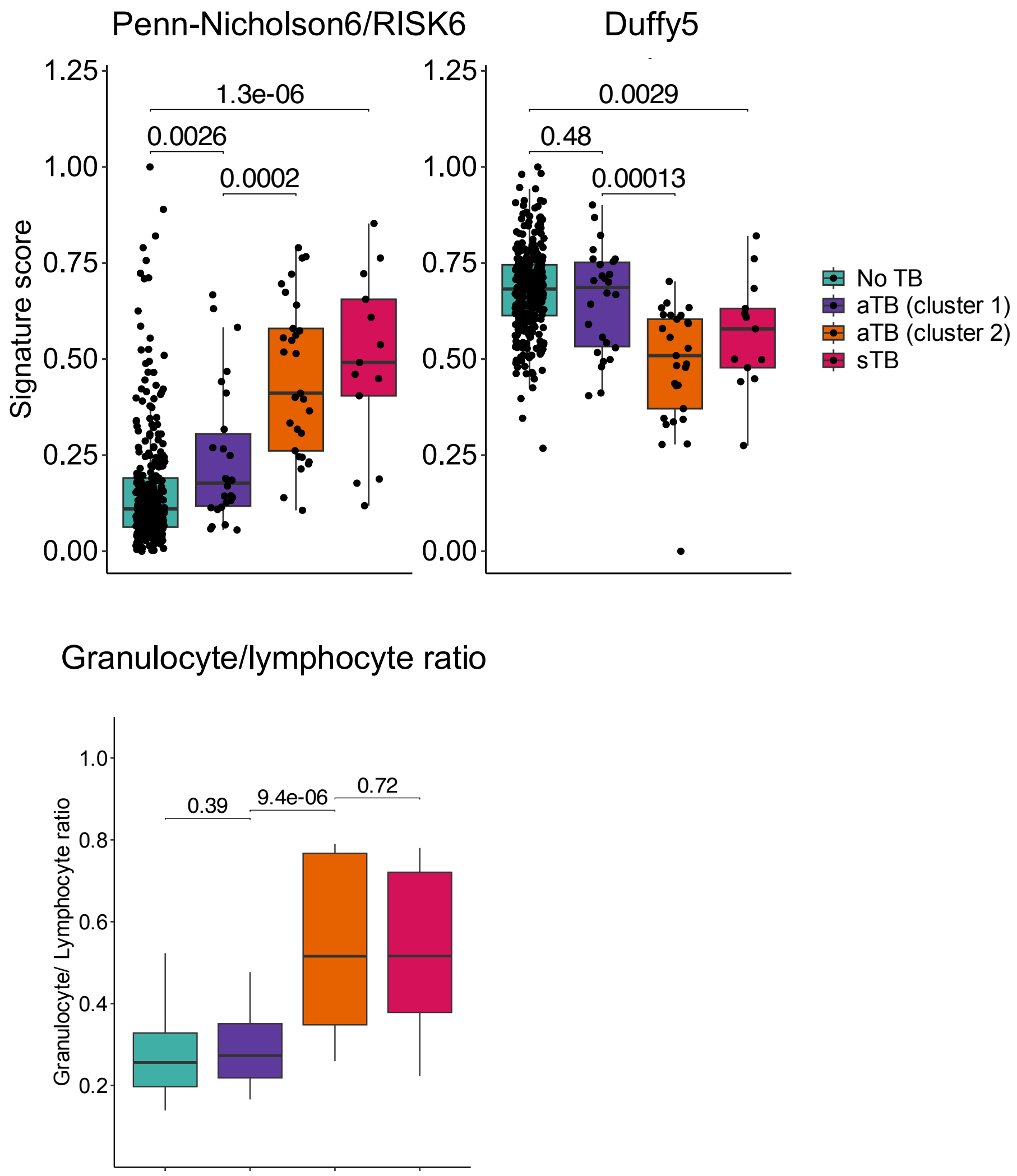


**Supplementary Figure 9. Transcriptomic signature scores for Penn-Nicholson6 (or RISK6), Duffy5 and Granulocyte/lymphocyte ratio in cluster 1 (n=26) and 2 (n=28) individuals as well as No-TB controls (n=321) or symptomatic TB (n=13).** The shown $p$-values were computed with the Mann-Whitney U test. For box-and-whisker plots horizontal lines indicate the median, the boxes the interquartile range (IQR) and the whiskers the range of data with $1.5 \times IQR$ from the lower and upper quartiles.

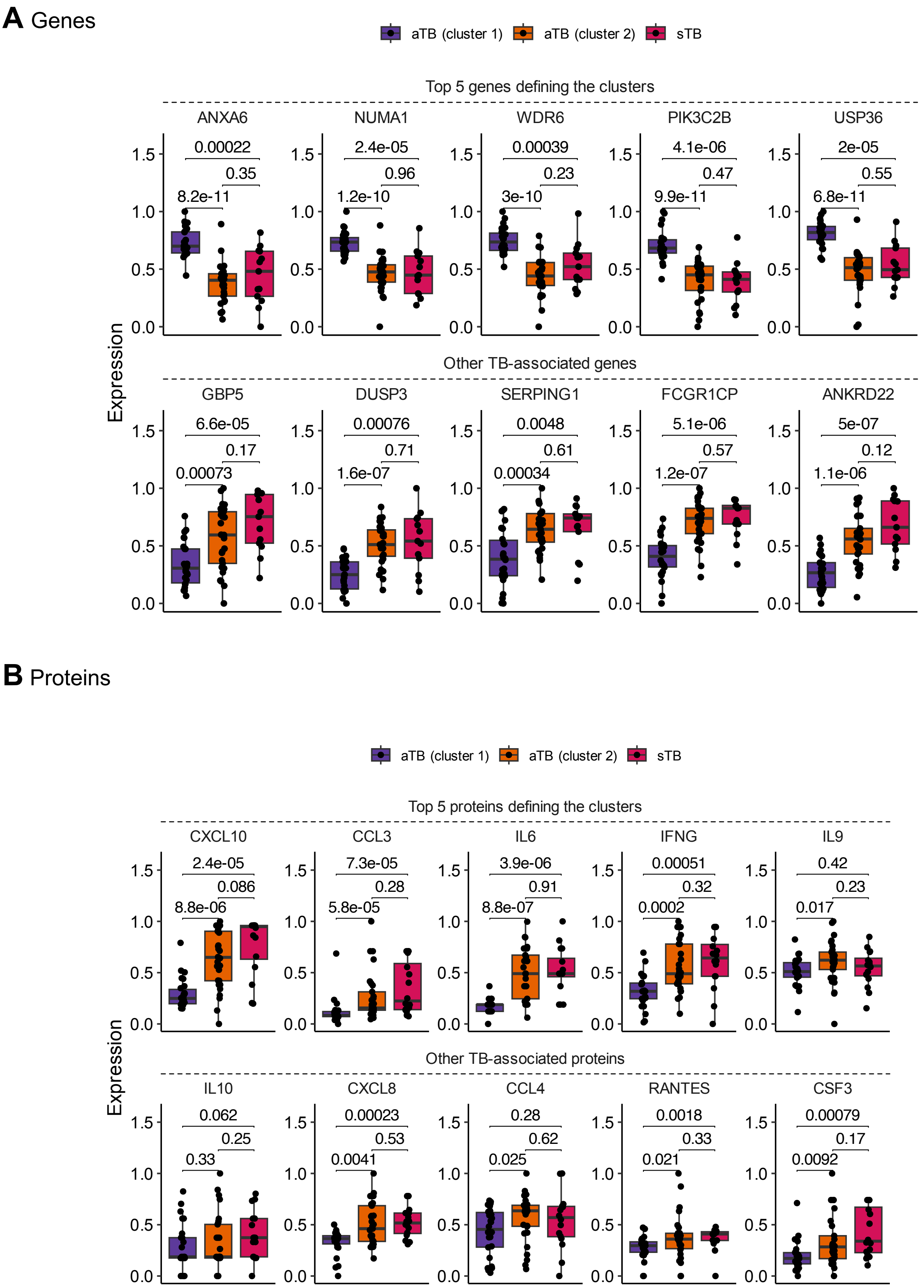


**Supplementary Figure 10. Analysis of expression levels of the top genes and proteins defining the two asymptomatic TB clusters obtained from fusion similarity network analysis.** (**A**) Expression levels of the top 5 genes defining the subclusters (top), contrasted with the expression levels of selected 5 known TB-associated genes (aTB cluster 1, n=26; aTB cluster 2, n=28; symptomatic TB, n=13). (**B**) Expression levels of the top 5 proteins defining the subclusters of asymptomatic TB (aTB) (top), contrasted with the expression levels of other selected 5 proteins

measured in the samples (aTB cluster 1, n=26; aTB cluster 2, n=28; symptomatic TB, n=13). $p$-values were obtained using Mann-Whitney U test.

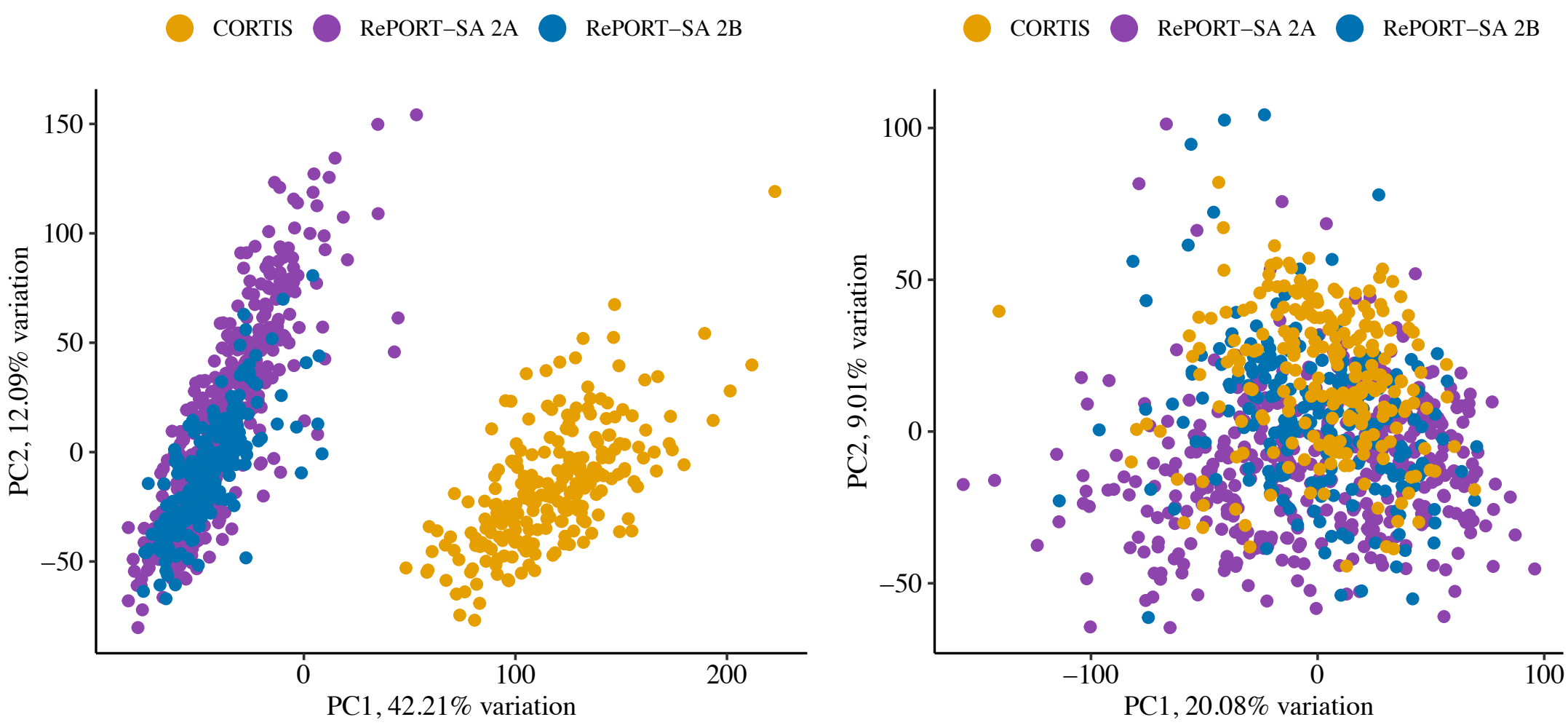


**Supplementary Figure 11. Principal component analysis before and after batch correction.** Principal component analysis (PCA) of merged normalized gene expression data from Clinic Triage (RePORT-SA 2A: no TB, n=212; symptomatic TB, n=197), HHC Screening (RePORT-SA 2B: no TB, n=144; asymptomatic TB, n=46; symptomatic TB, n=7), and Community Screening (CORTIS: no TB, n=180; asymptomatic TB, n=27; symptomatic TB, n=6) cohorts prior to batch adjustment (left), and after batch correction using ComBat-seq (right). Samples are colored by cohort. Clear clustering by cohort is observed along the first two principal components, indicating the presence of batch-associated technical variation across datasets. After adjustment, samples no longer cluster according to cohort, demonstrating effective reduction of batch-associated technical variation.

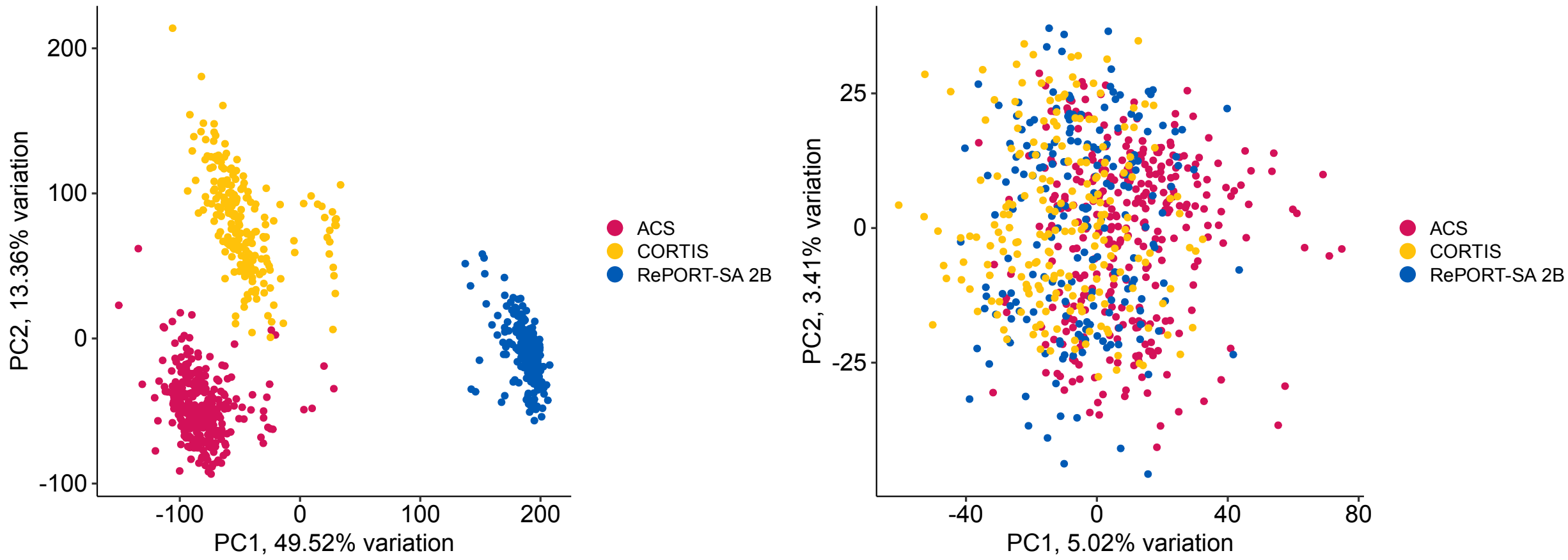


**Supplementary Figure 12. Principal component analysis before and after batch correction, to incorporate the ACS progressor and non-progressor subset.** Principal component analysis (PCA) of merged normalized gene expression data from HHC Screening (RePORT-SA 2B: no TB, n=144; asymptomatic TB, n=46; symptomatic TB, n=7), Community Screening (CORTIS: no TB, n=180; asymptomatic TB, n=27; symptomatic TB, n=6), and the Adolescent Cohort Study (ACS) progressor (n=76) and non-progressor (n=99) subset cohorts prior to batch adjustment (left), and

after batch correction using ComBat-seq (right). Samples are colored by cohort. Clear clustering by cohort is observed along the first two principal components, indicating the presence of batch-associated technical variation across datasets. After adjustment, samples no longer cluster according to cohort, demonstrating effective reduction of batch-associated technical variation.

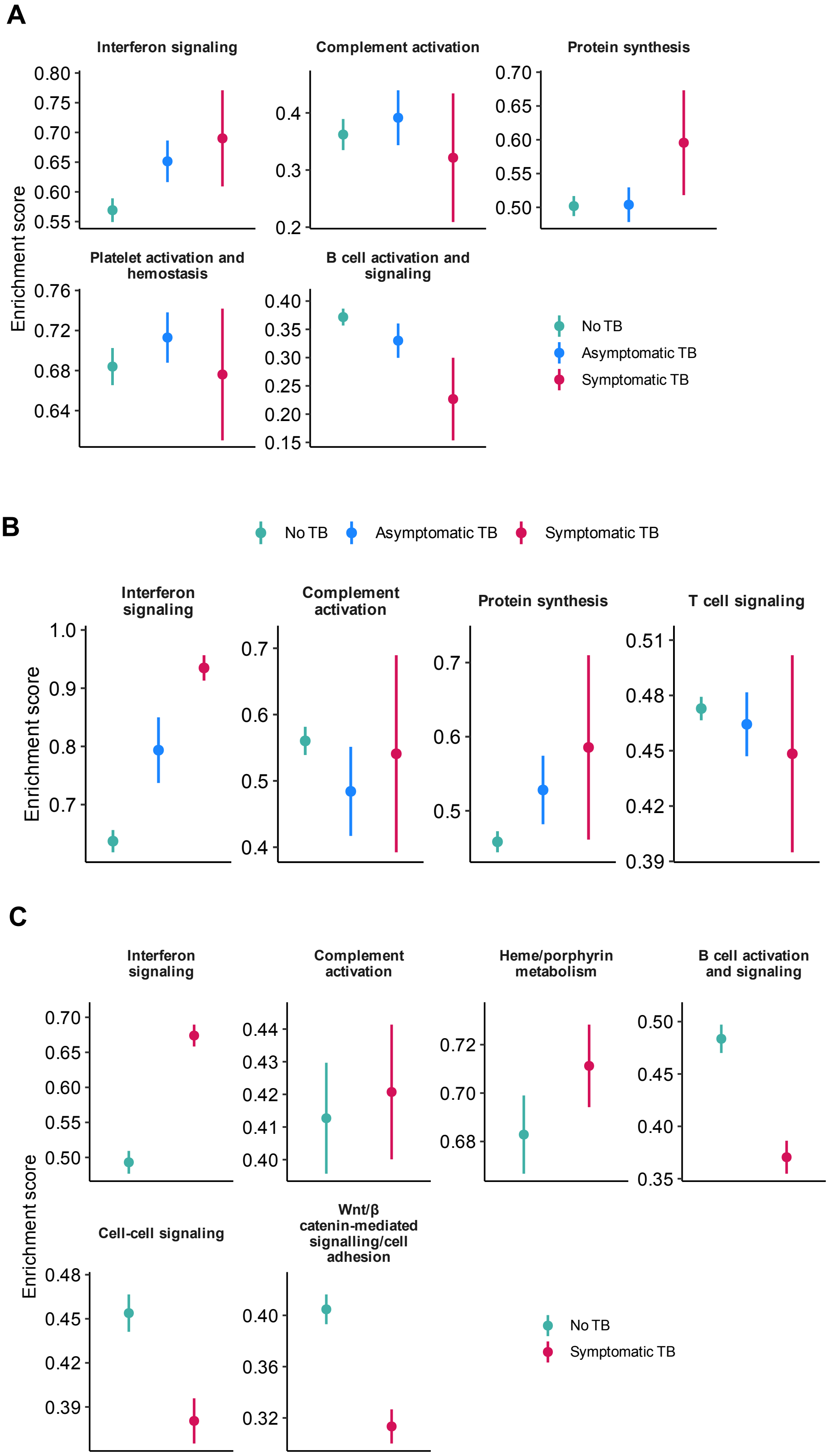


**Supplementary Figure 13. Cohort-specific WGCNA module enrichment scores across the TB disease spectrum.** Module enrichment scores for modules with significant pathway annotation

across TB disease groups for each cohort analysed independently: HHC Screening cohort (A: no TB, n=144; asymptomatic TB, n=46; symptomatic TB, n=7), Community Screening cohort (B: no TB, n=180; asymptomatic TB, n=27; symptomatic TB, n=6), and Clinic Triage cohort (C: no TB, n=212; symptomatic TB, n=197). Module numbers are not shown because performing WGCNA independently for the different cohorts yields inconsistent module numbering. Consistent trends in enrichment scores for annotated modules across the TB spectrum were observed within each cohort.

**References**


1. Zhang, Y., Parmigiani, G. & Johnson, W.E. ComBat-seq: batch effect adjustment for RNA-seq count data. *NAR Genom Bioinform* **2**, lqaa078 (2020).

2. Law, C.W., Chen, Y., Shi, W. & Smyth, G.K. voom: Precision weights unlock linear model analysis tools for RNA-seq read counts. *Genome Biol* **15**, R29 (2014).

3. Russo, P.S.T. *et al.* CEMiTool: a Bioconductor package for performing comprehensive modular co-expression analyses. *BMC Bioinformatics* **19**, 56 (2018).

4. Zak, D.E. *et al.* A blood RNA signature for tuberculosis disease risk: a prospective cohort study. *The Lancet* **387**, 2312–2322 (2016).

5. Scriba, T.J. *et al.* Sequential inflammatory processes define human progression from M. tuberculosis infection to tuberculosis disease. *PLoS Pathog* **13**, e1006687 (2017).

6. Altmann, A., Tolosi, L., Sander, O. & Lengauer, T. Permutation importance: a corrected feature importance measure. *Bioinformatics* **26**, 1340–1347 (2010).

7. Lundberg, S.M. *et al.* From Local Explanations to Global Understanding with Explainable AI for Trees. *Nat Mach Intell* **2**, 56–67 (2020).

8. Wang, B. *et al.* Similarity network fusion for aggregating data types on a genomic scale. *Nat Methods* **11**, 333–337 (2014).

9. Brock, G., Pihur, V., Datta, S. & Datta, S. clValid: An R Package for Cluster Validation. *Journal of Statistical Software* **25**, 1 – 22 (2008).

10. Yang, M. *et al.* Multi-omic integration via similarity network fusion to detect molecular subtypes of ageing. *Brain Communications* **5** (2023).
