# Supplementary material for: Inflammatory biomarkers of asymptomatic and symptomatic tuberculosis
Source: Nat Commun. 2026 Jul 25;17:9079. doi: 10.1038/s41467-026-75909-6 (PMC13518882; doi:10.1038/s41467-026-75909-6)
Supplement: Supplementary file 2 [file 41467_2026_75909_MOESM2_ESM.pdf]

nature portfolio

Corresponding author(s): Thomas Scriba

Last updated by author(s): May 5, 2026

Reporting Summary

Nature Portfolio wishes to improve the reproducibility of the work that we publish. This form provides structure for consistency and transparency in reporting. For further information on Nature Portfolio policies, see our Editorial Policies and the Editorial Policy Checklist.

Statistics

For all statistical analyses, confirm that the following items are present in the figure legend, table legend, main text, or Methods section.

n/a	Confirmed	
☐	☒	The exact sample size (*n*) for each experimental group/condition, given as a discrete number and unit of measurement
☐	☒	A statement on whether measurements were taken from distinct samples or whether the same sample was measured repeatedly
☐	☒	The statistical test(s) used AND whether they are one- or two-sided<br>*Only common tests should be described solely by name; describe more complex techniques in the Methods section.*
☐	☒	A description of all covariates tested
☐	☒	A description of any assumptions or corrections, such as tests of normality and adjustment for multiple comparisons
☐	☒	A full description of the statistical parameters including central tendency (e.g. means) or other basic estimates (e.g. regression coefficient) AND variation (e.g. standard deviation) or associated estimates of uncertainty (e.g. confidence intervals)
☐	☒	For null hypothesis testing, the test statistic (e.g. *F*, *t*, *r*) with confidence intervals, effect sizes, degrees of freedom and *P* value noted<br>*Give P values as exact values whenever suitable.*
☒	☐	For Bayesian analysis, information on the choice of priors and Markov chain Monte Carlo settings
☒	☐	For hierarchical and complex designs, identification of the appropriate level for tests and full reporting of outcomes
☐	☒	Estimates of effect sizes (e.g. Cohen's *d*, Pearson's *r*), indicating how they were calculated

Our web collection on statistics for biologists contains articles on many of the points above.

Software and code

Policy information about availability of computer code

Data collection	Participant-level data for the RePORT South Africa cohort were captured electronically using REDCap (version 15.0.38). Data for the CORTIS trial were collected using the Nukleus electronic data capture system (version 1.2).
Data analysis	Data were analyzed using R (version 4.3.3). Analysis code is available in a public GitHub repository (https://github.com/SATVILab/OmicScan; DOI: 10.5281/zenodo.2004196).

For manuscripts utilizing custom algorithms or software that are central to the research but not yet described in published literature, software must be made available to editors and reviewers. We strongly encourage code deposition in a community repository (e.g. GitHub). See the Nature Portfolio guidelines for submitting code & software for further information.

Data

Policy information about availability of data

All manuscripts must include a data availability statement. This statement should provide the following information, where applicable:

- Accession codes, unique identifiers, or web links for publicly available datasets
- A description of any restrictions on data availability
- For clinical datasets or third party data, please ensure that the statement adheres to our policy

Deidentified, processed gene expression data and raw fast data has been deposited on GEO repository (accession PRJNA1364747). Luminex data is available on ZivaHub at https://figshare.com/s/627c2e24299911aa77ca. Other source data are provided in the supplementary sections.

Research involving human participants, their data, or biological material

Policy information about studies with human participants or human data. See also policy information about sex, gender (identity/presentation), and sexual orientation and race, ethnicity and racism.

Reporting on sex and gender	Sex information was collected for all participants in the cohort studies. Sex was self-reported by participants during study enrollment.
Reporting on race, ethnicity, or other socially relevant groupings	Ethnicity information was collected for all study participants. Ethnicity was self-reported during study enrollment, and no additional verification or reclassification was performed.
Population characteristics	Key population characteristics were recorded and reported, including age, body mass index (BMI), prior tuberculosis history, study site, HIV status, sex, ethnicity, and smoking status.
Recruitment	Participants were recruited through cohort-specific enrollment procedures tailored to the respective study designs. These recruitment methods, including eligibility criteria and enrollment workflows, are described comprehensively in the Methods section.
Ethics oversight	The RePORT SA and CORTIS study protocols were approved by the institutional research ethics committees at each participating site; the RePORT SA protocol was also approved at Vanderbilt University Medical Center, USA. All participants provided written, informed consent prior to participation.

Note that full information on the approval of the study protocol must also be provided in the manuscript.

Field-specific reporting

Please select the one below that is the best fit for your research. If you are not sure, read the appropriate sections before making your selection.

☒ Life sciences ☐ Behavioural & social sciences ☐ Ecological, evolutionary & environmental sciences

For a reference copy of the document with all sections, see nature.com/documents/nr-reporting-summary-flat.pdf

Life sciences study design

All studies must disclose on these points even when the disclosure is negative.

Sample size	Sample sizes were determined by the predefined clinical design and enrollment frameworks of each cohort study. These sample size decisions were based on feasibility, and study objectives, and are described in detail in the manuscript.
Data exclusions	All data exclusions, including the criteria used and the number of participants or samples excluded, are detailed in the flow diagram presented in Figure 1 of the manuscript.
Replication	Not applicable
Randomization	Not applicable
Blinding	Not applicable.

Reporting for specific materials, systems and methods

We require information from authors about some types of materials, experimental systems and methods used in many studies. Here, indicate whether each material, system or method listed is relevant to your study. If you are not sure if a list item applies to your research, read the appropriate section before selecting a response.

Materials & experimental systems

n/a	Involved in the study	
☒	☐	Antibodies
☒	☐	Eukaryotic cell lines
☒	☐	Palaeontology and archaeology
☒	☐	Animals and other organisms
☐	☒	Clinical data
☒	☐	Dual use research of concern
☒	☐	Plants

Methods

n/a	Involved in the study	
☒	☐	ChIP-seq
☒	☐	Flow cytometry
☒	☐	MRI-based neuroimaging

Clinical data

Policy information about clinical studies

All manuscripts should comply with the ICMJE guidelines for publication of clinical research and a completed CONSORT checklist must be included with all submissions.

Clinical trial registration	The CORTIS clinical trial was prospectively registered at ClinicalTrials.gov under the identifier NCT02735590.
Study protocol	The study protocols for the CORTIS trial and RePORT South Africa are publicly available in the primary publications cited in this manuscript.
Data collection	Data collection sites for the CORTIS trial and the RePORT South Africa study are detailed in the primary publications cited.
Outcomes	Not applicable.

Plants

Seed stocks	Not applicable.
Novel plant genotypes	Not applicable.
Authentication	Not applicable.
