# Supplementary material for: Inflammatory biomarkers of asymptomatic and symptomatic tuberculosis
Source: Nat Commun. 2026 Jul 25;17:9079. doi: 10.1038/s41467-026-75909-6 (PMC13518882; doi:10.1038/s41467-026-75909-6)
Supplement: Supplementary file 3 [file 41467_2026_75909_MOESM3_ESM.pdf]

nature portfolio

Peer Review File

Inflammatory Biomarkers of Asymptomatic and Symptomatic Tuberculosis

Corresponding Author: Professor Thomas Scriba

This manuscript has been previously reviewed at another journal. This document only contains information relating to versions considered at Nature Communications.

This file contains all reviewer reports in order by version, followed by all author rebuttals in order by version.

Version 0:

Reviewer comments:

Reviewer #1

(Remarks to the Author)
The authors have addressed the reviewer comments appropriately. The epidemiological and statistical issues have been largely addressed.
I support acceptance of these revisions.

(Remarks on code availability)
Not applicable

Reviewer #3

(Remarks to the Author)
The authors addressed most of my comments except the one suggestion of integrating and benchmarking with rich publicly available single-cell TB datasets. The authors commented on "we feel that it is beyond the scope of the current work." I still think integrating or at least performing some meta-analyses of the key signatures and results from this work using the public single-cell data will benefit a lot, which will not take many months. For example, such meta-analysis can enhance the interpretation of the signatures learned from this study, yield infected or inflammatory cell state, immunology mechanism, and disease timeline through such analyses.

(Remarks on code availability)
No

Open Access This Peer Review File is licensed under a Creative Commons Attribution 4.0 International License, which permits use, sharing, adaptation, distribution and reproduction in any medium or format, as long as you give appropriate credit to the original author(s) and the source, provide a link to the Creative Commons license, and indicate if changes were made.
In cases where reviewers are anonymous, credit should be given to 'Anonymous Referee' and the source.
The images or other third party material in this Peer Review File are included in the article's Creative Commons license, unless indicated otherwise in a credit line to the material. If material is not included in the article's Creative Commons license and your intended use is not permitted by statutory regulation or exceeds the permitted use, you will need to obtain permission directly from the copyright holder.
To view a copy of this license, visit https://creativecommons.org/licenses/by/4.0/

Reviewer 3 comment:

There are several comprehensive single-cell transcriptomics and proteomics analyses which revealed key T cell states and signatures, and myeloid pathways related to latent TB. The cell type and mechanism information is missing from this current study. Integration and benchmarking using publicly available single-cell datasets would help yield fine-grained cell state information and new knowledge. For example, Nathan, et al, Multimodally profiling memory T cells from a tuberculosis cohort identifies cell state associations with demographics, environment and disease, Nature Immunology, 2021; Sun, et al, A single-cell transcriptomic atlas reveals senescence and inflammation in the post-tuberculosis human lung, Nature microbiology, 2025

Response: Reviewer 3 requested that we perform additional analyses by accessing publicly available single-cell TB datasets from Nathan et al., Nat Immunol. 2021 and Sun Nature Microb. 2025. It is not clear to us how doing the suggested new analyses using the single cell RNAseq datasets reported in the two studies will be useful or fit into the broad interpretations of our study. The cohorts from these two studies do not include participants with asymptomatic TB. Rather, Nathan et al., collected samples from people with household contact who previously had TB and Sun et al., performed spatial RNAseq on TB lesions from lungs of people who previously had TB (post-TB lungs).

In light of this, we want to reiterate that any extra effort to perform analyses using these datasets, which are not from people with asymptomatic TB and therefore do not address an obvious scientific question that relates to the findings in our submitted manuscript, would require significant effort at the risk of yielding results that do not relate to biological underpinnings of asymptomatic TB.
